# The LRXs-RALFs-FER module controls plant growth and salt stress responses by modulating multiple plant hormones

**DOI:** 10.1093/nsr/nwaa149

**Published:** 2020-06-30

**Authors:** Chunzhao Zhao, Wei Jiang, Omar Zayed, Xin Liu, Kai Tang, Wenfeng Nie, Yali Li, Shaojun Xie, Yuan Li, Tiandan Long, Linlin Liu, Yingfang Zhu, Yang Zhao, Jian-Kang Zhu

**Affiliations:** Shanghai Center for Plant Stress Biology and Center of Excellence in Molecular Plant Sciences, Chinese Academy of Sciences, Shanghai 200032, China; Department of Horticulture and Landscape Architecture, Purdue University, West Lafayette, IN 47907, USA; Key laboratory of Plant Stress Biology, School of Life Sciences, Henan University, Kaifeng 475004, China; Department of Horticulture and Landscape Architecture, Purdue University, West Lafayette, IN 47907, USA; National Center for Soybean Improvement, Key Laboratory of Biology and Genetics and Breeding for Soybean, Ministry of Agriculture, State Key Laboratory of Crop Genetics and Germplasm Enhancement, Nanjing Agricultural University, Nanjing 210095, China; Department of Horticulture and Landscape Architecture, Purdue University, West Lafayette, IN 47907, USA; Genetics Department, Faculty of Agriculture, Menofia University, Shebeen Elkoum 32511, Egypt; Shanghai Center for Plant Stress Biology and Center of Excellence in Molecular Plant Sciences, Chinese Academy of Sciences, Shanghai 200032, China; University of the Chinese Academy of Sciences, Beijing 100049, China; Shanghai Center for Plant Stress Biology and Center of Excellence in Molecular Plant Sciences, Chinese Academy of Sciences, Shanghai 200032, China; Department of Horticulture and Landscape Architecture, Purdue University, West Lafayette, IN 47907, USA; Shanghai Center for Plant Stress Biology and Center of Excellence in Molecular Plant Sciences, Chinese Academy of Sciences, Shanghai 200032, China; Shanghai Center for Plant Stress Biology and Center of Excellence in Molecular Plant Sciences, Chinese Academy of Sciences, Shanghai 200032, China; Department of Horticulture and Landscape Architecture, Purdue University, West Lafayette, IN 47907, USA; Department of Horticulture and Landscape Architecture, Purdue University, West Lafayette, IN 47907, USA; State Key Laboratory of Plant Physiology and Biochemistry, College of Biological Sciences, China Agricultural University, Beijing 100193, China; Department of Horticulture and Landscape Architecture, Purdue University, West Lafayette, IN 47907, USA; State Key Laboratory of Crop Gene Exploration and Utilization in Southwest China, Sichuan Agricultural University, Chengdu 611130, China; Shanghai Center for Plant Stress Biology and Center of Excellence in Molecular Plant Sciences, Chinese Academy of Sciences, Shanghai 200032, China; University of the Chinese Academy of Sciences, Beijing 100049, China; Key laboratory of Plant Stress Biology, School of Life Sciences, Henan University, Kaifeng 475004, China; Shanghai Center for Plant Stress Biology and Center of Excellence in Molecular Plant Sciences, Chinese Academy of Sciences, Shanghai 200032, China; Key laboratory of Plant Stress Biology, School of Life Sciences, Henan University, Kaifeng 475004, China; Shanghai Center for Plant Stress Biology and Center of Excellence in Molecular Plant Sciences, Chinese Academy of Sciences, Shanghai 200032, China; Department of Horticulture and Landscape Architecture, Purdue University, West Lafayette, IN 47907, USA

**Keywords:** salt stress, cell wall integrity, growth, LRX, RALF, FER, hormone

## Abstract

Salt stress is a major environmental factor limiting plant growth and productivity. We recently discovered an important new salt tolerance pathway, where the cell wall leucine-rich repeat extensins LRX3/4/5, the RAPID ALKALINIZATION FACTOR (RALF) peptides RALF22/23 and receptor-like kinase FERONIA (FER) function as a module to simultaneously regulate plant growth and salt stress tolerance. However, the intracellular signaling pathways that are regulated by the extracellular LRX3/4/5-RALF22/23-FER module to coordinate growth, cell wall integrity and salt stress responses are still unknown. Here, we report that the LRX3/4/5-RALF22/23-FER module negatively regulates the levels of jasmonic acid (JA), salicylic acid (SA) and abscisic acid (ABA). Blocking JA pathway rescues the dwarf phenotype of the *lrx345* and *fer-4* mutants, while disruption of ABA biosynthesis suppresses the salt-hypersensitivity of these mutants. Many salt stress-responsive genes display abnormal expression patterns in the *lrx345* and *fer-4* mutants, as well as in the wild type plants treated with epigallocatechin gallate (EGCG), an inhibitor of pectin methylesterases, suggesting cell wall integrity as a critical factor that determines the expression pattern of stress-responsive genes. Production of reactive oxygen species (ROS) is constitutively increased in the *lrx345* and *fer-4* mutants, and inhibition of ROS accumulation suppresses the salt-hypersensitivity of these mutants. Together, our work provides strong evidence that the LRX3/4/5-RALF22/23-FER module controls plant growth and salt stress responses by regulating hormonal homeostasis and ROS accumulation.

## INTRODUCTION

Leucine-rich repeat extensins (LRXs) are cell wall-localized proteins important for the regulation of cell wall integrity. In Arabidopsis, there are 11 LRX proteins, named LRX1-LRX11 [1,2]. Mutations in *LRX8*, *LRX9* and *LRX11* lead to altered compositions of extensins, arabinogalactan proteins, rhamnogalacturonan I and xyloglucan in the cell wall of pollen tubes [3,4]. It has also been reported that callose in the apical walls of pollen tubes accumulates in the *lrx8 lrx9 lrx11* and *lrx9 lrx10 lrx11* mutants [3–5]. In the *lrx3 lrx4 lrx5* (hereafter referred to as *lrx345*) triple mutant, the amounts of rhamnose, galactose, arabinogalactan proteins, extensins and arabinan are reduced, while the levels of mannose and lignin are increased compared with the wild type [6]. The mechanisms by which LRX proteins regulate the deposition of cell wall components remain to be elucidated.

LRX proteins harbor an N-terminal LRR domain and a C-terminal extensin domain, both of which function in the extracellular region [2,7]. The lack of an intracellular domain suggests that LRX proteins need partners to transduce cell wall signals to the cell interior. LRX8-LRX11 physically interact with RALF4/19 in the cell wall of pollen tubes and feed into the ANX1/2-mediated signaling pathway, and thus regulate pollen germination and pollen tube growth [8,9]. The binding of LRXs with RALF4 has been confirmed by a recent structural study, which showed that these two components exhibit a strong affinity and LRX proteins preferentially bind folded RALF peptides [10]. It has also been shown that RALF4/19 are ligands for ANX1/2 and BUPS1/2 [11]. Our previous study showed that LRX3, LRX4 and LRX5 function together with RALF22/23 and FER to regulate plant growth and salt stress responses [12]. FER is a plasma membrane kinase that acts as a receptor of several RALF peptides [13,14]. Recently, it was reported that THE1 is a receptor for RALF34 and the THE1-RALF34 module is required for the regulation of lateral root initiation [15]. Notably, ANX1/2, FER and THE1 all belong to the *Catharanthus roseus* receptor-like kinase 1-like (*Cr*RLK1L) protein family [9]. Based on these data, we can speculate that LRXs, RALFs and *Cr*RLK1L family proteins function as modules to monitor cell wall status and transduce cell wall signals and regulate cell wall integrity in different tissues or upon exposure to different environmental stresses.

The *lrx345* and *fer-4* mutants as well as transgenic plants overexpressing *RALF22* or *RALF23* display similar phenotypes, including dwarfism, salt-hypersensitivity, increased accumulation of anthocyanin and increased susceptibility to bacteria [12,14]. It was proposed that FER regulates cell expansion via phosphorylation of the proton pump AHA2 [13,16], although genetic evidence for this is still lacking. FER positively regulates the phosphatase activity of ABI2, a negative regulator of the ABA core signaling pathway, and thus negatively modulates ABA sensitivity [17]. FER directly binds with pectins and is required for the activation of downstream cell wall repair pathways via Ca^2+^-mediated signals under salt stress [18]. The reduced root elongation of the *fer* mutant under salt stress could be caused by impaired cell wall integrity, which results in dramatic root cell burst during growth recovery [18]. FER is also involved in the positive regulation of plant immunity through the regulation of the association of the FLS2/EFR-BAK1 complex and the stability of MYC2 [14,19].

So far, the mechanisms underlying the enhanced cell death of the *lrx345* and *fer* mutants under high salinity remain unknown. Here, we investigated intracellular signaling pathways that are regulated by the LRX3/4/5-RALF22/23-FER module. Our results show that three hormones, JA, SA and ABA, are consitutively increased in the *lrx345* and *fer* mutants, and that elevated levels of these hormones are responsible for the dwarf phenotype and salt-hypersensitivity of the *lrx345* and *fer-4* mutants. Our data also reveal that LRX3/4/5-RALF22/23-FER module-mediated cell wall integrity signals play a crucial role in determining the expression of stress-responsive genes after both short and prolonged exposure to high salinity.

## RESULTS

### Jasmonic acid (JA)- and salicylic acid (SA)-responsive genes are constitutively up-regulated in the *lrx345* triple mutant

**Figure 1.**
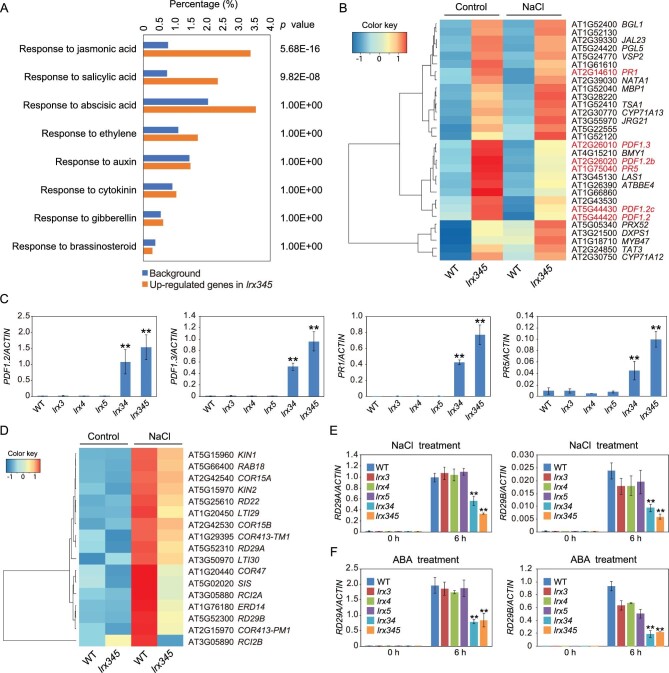
Transcriptome profiles of wild type and *lrx345* mutant plants before and after NaCl treatment. (A) GO enrichment analysis of genes that were up-regulated in the *lrx345* mutant compared with the wild type under normal conditions. The enrichment of genes responsive to eight plant hormones are shown. (B) Heat map of the 30 genes that were up-regulated with highest fold changes in the *lrx345* mutant plants compared with the wild type. *PDF* and *PR* genes are indicated in red. (C) qRT-PCR analysis of the transcript levels of *PDF1.2*, *PDF1.3*, *PR1* and *PR5* in wild type and *lrx* single, double and triple mutants. *ACTIN8* was used as an internal control. Values are means of three biological replicates ± SD, **P < 0.01 (Student's *t*-test). (D) Heat map of the abiotic stress-responsive genes that exhibited lower transcript levels in the *lrx345* mutant compared to wild type after NaCl treatment for 6 h. (E, F) qRT-PCR analysis of the gene expression of *RD29A* and *RD29B* in the indicated mutant plants before and after NaCl (150 mM) (E) or ABA (50 μM) (F) treatment for 6 h. *ACTIN8* was used as an internal control. Values are means of three biological replicates ± SD, **P < 0.01 (Student's *t*-test).

To gain insight into the mechanisms underlying the dwarf phenotype and salt-hypersensitivity of the *lrx345* triple mutant [12], we performed RNA-seq analysis for the wild type and *lrx345* mutant seedlings before and after NaCl treatment for 6 h. We first compared gene expression between the wild type and *lrx345* mutant without NaCl treatment. In total, 1418 genes were significantly up-regulated (fold change >2, *P* value < 0.05) and 1609 genes were significantly down-regulated (fold change >2, *P* value < 0.05) in the *lrx345* mutant (Table S1). Gene ontology (GO) enrichment analysis showed that genes belonging to categories ‘response to jasmonic acid’ (*P* value = 5.68E-16) and ‘response to salicylic acid’ (*P* value = 9.82E-08) were significantly enriched among the up-regulated genes (Fig. 1A). Specifically, 34.2% of JA-responsive genes, including *PDF1.2*, *PDF1.2b*, *PDF1.2c*, *PDF1.3* and *VSP2*, and 30.8% of SA-responsive genes, including *PR1* and *PR5*, were constitutively up-regulated in the *lrx345* mutant (Fig. 1B). Quantitative real-time (qRT)-PCR analysis showed that the increased expression of *PDF* and *PR* genes occurred only in the *lrx34* double and *lrx345* triple mutants but not in any of the *lrx* single mutants, suggesting that the three LRX proteins are redundant in the regulation of JA and SA pathways (Fig. 1C). By contrast, genes responsive to other hormones, including abscisic acid, ethylene, auxin, cytokinin, gibberellin and brassinosteroid, were not significantly enriched (*P* value < 0.05) among the up-regulated genes (Fig. 1A). These results indicate that LRX3/4/5 are involved in the negative regulation of JA and SA signaling pathways.

### Genes involved in abiotic stress response and cell wall modification display lower transcript levels in the *lrx345* mutant after salt treatment

Comparison of gene expression between the wild type and *lrx345* mutant after NaCl treatment showed that 1330 genes were up-regulated and 575 genes were down-regulated in the *lrx345* mutant (Table S2). As under normal conditions, up-regulated genes were enriched in the categories ‘response to jasmonic acid’ (*P* value = 9.81E-21) and ‘response to salicylic acid’ (*P* value = 7.88E-13). Down-regulated genes were enriched in the categories ‘cell wall organization’ (*P* value = 9.89E-15), ‘cell wall modification’ (*P* value = 6.15E-05), ‘xyloglucan metabolic process’ (*P* value = 2.70E-04) and ‘hemicellulose metabolic process’ (*P* value = 1.30E-03) (Fig. S1A). The cell wall-related genes that are regulated by the LRX proteins include those encoding pectin lyase-like superfamily proteins, expansins, xyloglucan endotransglucosylase/hydrolases, casparian strip membrane domain proteins and glycosyl hydrolases (Fig. S1B). The down-regulation of these cell wall-related genes in the *lrx345* mutant under salt stress was confirmed by qRT-PCR analysis (Fig. S1C). The down-regulated genes were also enriched in the category ‘response to water deprivation’ (*P* value = 2.80E-04) (Fig. S1A). The genes belonging to this category include many well-known abiotic stress responsive genes, such as *RD29A*, *RD29B*, *RD22*, *RAD18*, *COR15A* and *KIN1* (Fig. 1D). qRT-PCR analysis confirmed that the expression of *RD29A* and *RD29B* genes was substantially down-regulated in the *lrx34* double and *lrx345* triple mutants, but was not significantly affected in the *lrx* single mutants after salt treatment compared with the wild type (Fig. 1E). Also ABA-induced up-regulation of *RD29A* and *RD29B* was attenuated in the *lrx345* mutant (Fig. 1F). Together, these results indicate that LRX3/4/5 proteins are involved in the regulation of abiotic stress-responsive genes and cell wall modification genes under salt stress.

Turgor pressure is important for cell expansion. Under high salinity, maintainance of turgor pressure requires the uptake of K^+^ and increased water flow to the cell [20]. From our RNA-seq data, we found that the expression of K^+^ transporters *AtHAK5* [21] and *KUP11* [22], was significantly decreased in the *lrx345* mutant under salt stress (Table S2). Interestingly, among 36 aquaporin genes that were investigated, none of them showed a significantly increased expression but 16 genes displayed significantly reduced expression in the *lrx345* mutant compared with the wild type under high salinity (Table S3). The results suggest that the LRX proteins are important for the regulation of turgor pressure and water homeostasis under salt stress.

### *PDF* and *PR* genes are highly up-regulated in the *fer-4* mutant and in transgenic plants overexpressing *RALF22*/*23*

**Figure 2.**
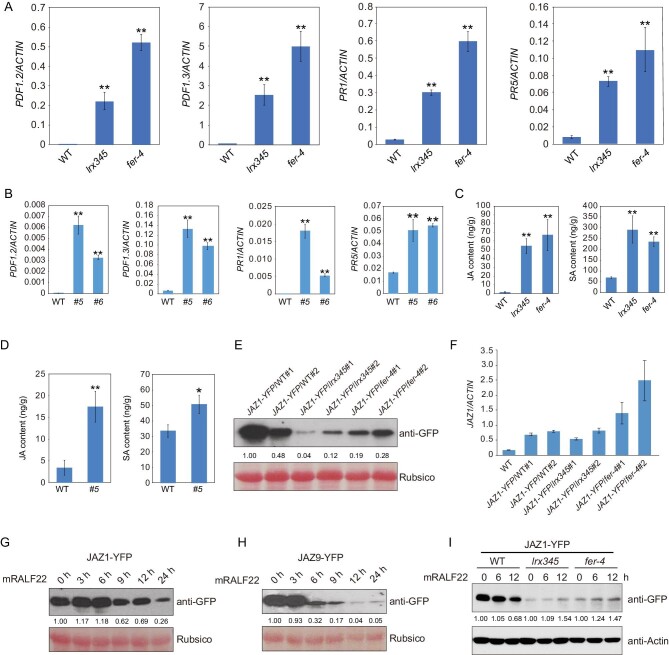
LRX3/4/5-RALF22/23-FER module negatively regulates JA signaling pathway. (A) qRT-PCR analysis of the transcript levels of *PDF1.2*, *PDF1.3*, *PR1* and *PR5* in wild type, *lrx345* and *fer-4* seedlings. *ACTIN8* was used as an internal control. (B) qRT-PCR analysis of the transcript levels of *PDF1.2*, *PDF1.3*, *PR1* and *PR5* in wild type and two independent *RALF22* overexpressing lines (*#5* and *#6*). *ACTIN8* was used as an internal control. (C) Jasmonic acid (JA) and salicylic acid (SA) contents in wild type, *lrx345* and *fer-4* seedlings. (D) JA and SA contents in transgenic plants overexpressing *RALF22*. (E) Immunoblotting analysis of JAZ1 protein in wild type, *lrx345* and *fer-4* seedlings expressing JAZ1-YFP. (F) qRT-PCR analysis of the *JAZ1* transcript levels in the transgenic plants. (G, H) Transgenic plants expressing *JAZ1-GFP* (G) and *JAZ9-GFP* (H) were treated with mature RALF22 (mRALF22) for 0, 3, 6, 9, 12 and 24 h. Immunoblottings were performed using anti-GFP antibody. (I) Ten-day-old seedlings were treated with mRALF22 (1 μM) for 0, 6 and 12 h. Immunoblotting assays were performed using anti-GFP and anti-Actin antibodies. The band intensity in (E), (G) and (H) was qualified using ImageJ software. Values in (A–D) are means of three biological replicates ± SD, *P < 0.05 and **P < 0.01 (Student's *t*-test).

The JA- and SA-responsive genes *PDF1.2*, *PDF1.3*, *PR1* and *PR5* were also constitutively up-regulated in the *fer-4* mutant and in transgenic plants overexpressing *RALF22* or *RALF23* (Fig. 2A and B, and Fig. S2A), consistent with previous evidence that LRX3/4/5, RALF22/23 and FER function in the same pathway [12]. To determine whether increased hormone accumulation underpins the up-regulation of JA- and SA-responsive genes, we measured the levels of JA and SA in the *lrx345* and *fer-4* mutants. In both mutants, the level of JA was more than 30-fold higher, while the level of SA was more than 3-fold higher, than that in the wild type (Fig. 2C). Transgenic plants overexpressing *RALF22* also showed higher levels of JA and SA than the wild type (Fig. 2D).

To understand the causes of JA and SA over-accumulation in the *lrx345* mutant, we analyzed the expression of genes involved in JA and SA biosynthesis in the RNA-seq data. Of the 25 genes involved in JA biosynthesis, 14 were increased by more than 2-fold in the *lrx345* mutant (Fig. S2B). qRT-PCR confirmed that JA biosynthesis-related genes were constitutively up-regulated in the *lrx345* and *fer-4* mutants (Fig. S2C). Both RNA-seq and qRT-PCR assays showed that *ICS1*, *EPS1*, *CBP60G* and *PBS3*, which are important for SA biosynthesis, were significantly up-regulated in the *lrx345* mutant (Fig. S3A and B). These results indicate that the elevated contents of JA and SA in the *lrx345* and *fer-4* mutants are caused by the up-regulation of JA- and SA-biosynthesis genes.

Accumulation of JA results in the degradation of JASMONATE ZIM-DOMAIN (JAZ) proteins [23]. To further elucidate the role of the LRX3/4/5-RALF22/23-FER module in regulating the JA signaling pathway, we generated transgenic plants expressing JAZ1-YFP in the wild type, *lrx345* and *fer-4* backgrounds. JAZ1 protein but not its transcript levels were markedly lower in the *lrx345* and *fer-4* mutants (Fig. 2E and F). Consistent with the result that overexpression of *RALF22* triggered the accumulation of JA (Fig. 2D), application of exogenous mature RALF22 (mRALF22) induced the degradation of JAZ1 (Fig. 2G). Similarly, mRALF22 also triggered the degradation of JAZ9 protein (Fig. 2H). To understand whether mRALF22-triggered degradation of JAZs is mediated by LRX3/4/5 and FER, we examined the protein level of JAZ1 in *lrx345* and *fer-4* mutants after mRALF22 treatment. We found that mRALF22 could not trigger JAZ1 degradation in the *lrx345* and *fer-4* mutants (Fig. 2I). Together, the above results indicate that the LRX3/4/5-RALF22/23-FER module negatively regulates the JA and SA signaling pathways.

### Disruption of the JA pathway suppresses the dwarf phenotype of the *lrx345* and *fer-4* mutants

**Figure 3.**
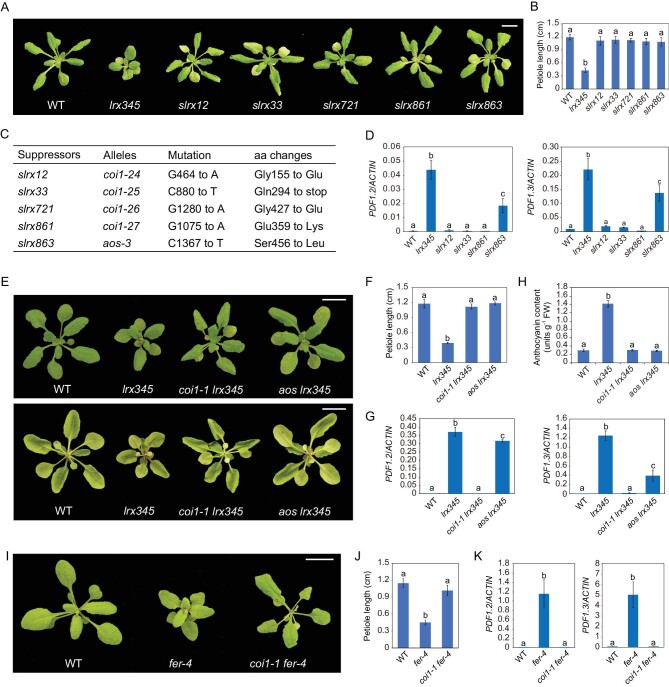
Disruption of the JA pathway suppresses the dwarf phenotype of the *lrx345* and *fer-4* mutants. (A) Rosette morphology of the wild type, *lrx345*, and five suppressors *of lrx345* (*slrx*) grown in soil. Scale bar, 1 cm. (B) Petiole length of each genotype grown in soil for 4 weeks. (C) Mutations identified in the *lrx* suppressors by whole-genome sequencing-based mapping. (D) qRT-PCR analysis of the transcript levels of *PDF1.2* and *PDF1.3*. *ACTIN8* was used as an internal control. (E) Morphology of the upper epidermis (upper panel) and lower epidermis (bottom panel) of plants grown in soil. Scale bar, 1 cm. (F) Petiole lengths of the wild type, *lrx345*, *coi1-1 lrx345* and *aos lrx345* grown in soil for 4 weeks. (G) qRT-PCR analysis of the transcript levels of *PDF1.2* and *PDF1.3*. *ACTIN8* was used as an internal control. (H) Quantification of anthocyanin accumulation in 12-day-old seedlings of the wild type, *lrx345*, *coi1-1 lrx345* and *aos lrx345*. (I) Rosette morphology of the wild type, *fer-4* and *coi1-1 fer-4*. Scale bar, 1 cm. (J) Petiole lengths of the wild type, *fer-4* and *coi1-1 fer-4* grown in soil for 4 weeks. (K) qRT-PCR analysis of the transcript levels of *PDF1.2* and *PDF1.3* in wild type, *fer-4* and *coi1-1 fer-4*. *ACTIN8* was used as an internal control. Values in (B), (F) and (J) are means of 10 petioles ± SD. Values in (D), (G), (H) and (K) are means of three biological replicates ± SD. Different letters represent statistically significant differences (*P* < 0.01, one-way ANOVA).

To identify downstream factors of the LRX3/4/5-RALF22/23-FER signaling module, we screened for the suppressors of *lrx345* (referred to as *slrx*) using ethyl methanesulfonate (EMS)-mediated mutagenesis. Approximately 15 000 *lrx345* seeds were used for EMS mutagenesis and 18 suppressors that could rescue the dwarf phenotype of the *lrx345* mutant were identified. As these suppressors displayed a similar growth phenotype (Fig. 3A and B), five of them were chosen for whole-genome sequencing-based mapping. We identified mutations in the *COI1* gene, encoding the JA receptor [24], in four of the five suppressors, while a mutation in the *AOS* gene, encoding a JA biosynthetic enzyme [25], was found in the remaining suppressor (Fig. 3C). Consistent with these results, we found that the expression of the JA-responsive genes *PDF1.2* and *PDF.3* was attenuated in the suppressors (Fig. 3D). By crossing *lrx345* to previously published *coi1-1* and *aos* mutants [24,25], we confirmed that mutations in these genes fully recovered the growth of the *lrx345* mutant (Fig. 3E and F), and also reduced the expression of *PDF* genes (Fig. 3G). It should be noted that the reduction of *PDF* genes expression was less pronounced in the *aos* mutant than that in the *coi1* mutant (Fig. 3D and G). The increased accumulation of anthocyanin in the *lrx345* mutant was also suppressed by the *AOS* and *COI1* mutations (Fig. 3E and H). In the *fer-4* mutant, the dwarf phenotype and the increased expression of *PDF* genes were similarly suppressed by the *coi1* mutation (Fig. 3I–K). These results indicate that constitutive activation of the JA pathway is responsible for the dwarfism and accumulation of anthocyanin in the *lrx345* and *fer-4* mutants.

We then tested whether disrupting the JA pathway can reverse the salt-hypersensitivity of *lrx345* and *fer-4* mutants. Both *coi1-1* and *aos* mutants are male-sterile [24,25], but the *aos* mutant can produce seeds when exogenous JA is applied, so we obtained *aos lrx345* homozygous plants and tested their response to salt stress. The *aos* mutation only slightly suppressed the salt-hypersensitivity of *lrx345* mutant (Fig. S4A and B). Further, we crossed the *coi1-16* mutant allele [26], which can produce seeds at low temperatures (16°C), with the *lrx345* mutant, and found that the *coi1-16* mutation also only slightly suppressed the salt-hypersensitivity of *lrx345* mutant (Fig. S4A and B).

The *jazQ* mutant, which lacks five JAZ transcriptional repressors, exhibits constitutive JA responses [27]. Similar to the *lrx345* mutant, the *jazQ* mutant displays a dwarf phenotype [27] (Fig. S4C and D). To determine whether constitutive activation of JA pathway results in enhanced cell death under salt stress, we grew the *jazQ* mutant on medium with a high concentration of salt. The *jazQ* mutant survived as well as the wild type under salt stress (Fig. S4E). Besides, mutation of *MYC2*, which encodes a key transcription factor of the JA signaling pathway, partially suppressed the dwarf phenotype, but only slightly suppressed the salt-hypersensitivity of the *lrx345* mutant (Fig. S4F–H). Together, these results indicate that activation of the JA pathway alone is not sufficient to cause cell death in plants under high salinity.

To assess the contribution of the increased SA content to the phenotypes of the *lrx345* mutant, we crossed *lrx345* to the *sid2-2* mutant, in which SA biosynthesis is highly impaired [28]. The mutation of *SID2* did not reverse the dwarf phenotype of *lrx345*, but partially suppressed its salt-hypersensitivity (Fig. S3C–E). As expected, the up-regulation of *PR1* was completely reversed in the *sid2-2 lrx345* quadruple mutant, while the expression of *PDF1.2* and *PDF1.3* was not significantly changed (Fig. S3F), indicating that the increased expression of *PR1* in the *lrx345* mutant depends on the SA pathway.

In our suppressor screen, we also identified three genes, *TT3*, *TT4* and *TT6*, that are required for the anthocyanin accumulation in the *lrx345* mutant, but mutations in these genes did not suppress the dwarf phenotype and salt-hypersensitivity of the *lrx345* mutant (Fig. S5). These three genes, encoding dihydroflavonol reductase, chalcone synthase and flavanone 3-hydroxylase, respectively, are required for anthocyanin biosynthesis in Arabidopsis [29–31]. Disruption of JA biosynthesis or perception suppresses both the anthocyanin accumulation and plant growth phenotypes of the *lrx345* mutant (Fig. 3E and H), but abolishing anthocyanin biosynthesis only affects the accumulation of anthocyanin, suggesting that JA signaling can feed forward into anthocyanin production.

### Salt-hypersensitivity of the *lrx345* and *fer-4* mutants is dependent on ABA

**Figure 4.**
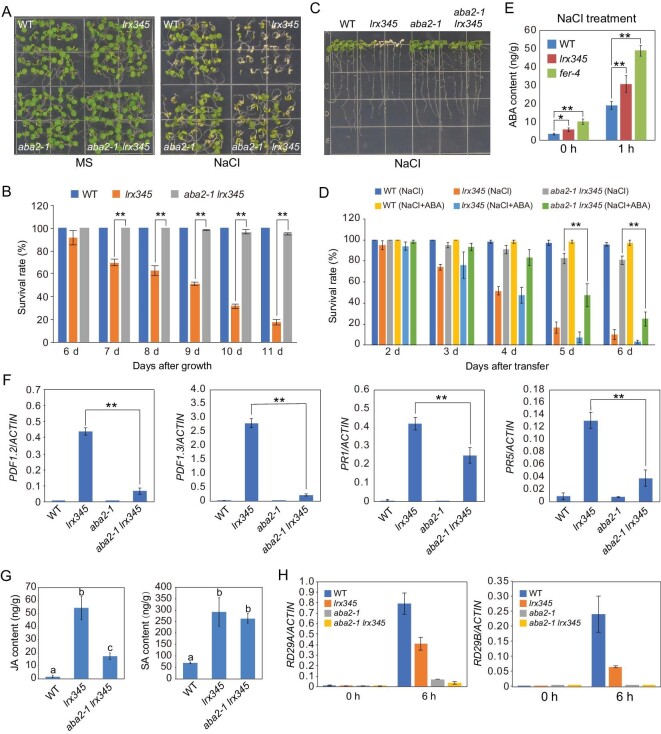
Suppression of the salt-hypersensitive phenotype of *lrx345* mutant plants by a mutation in *ABA2*. (A) Phenotypes of seedlings of the indicated genotypes grown on MS and MS+NaCl (120 mM) media. (B) Survival rates of the wild type, *lrx345* and *aba2-1 lrx345* seedlings grown on MS+NaCl (120 mM) medium. (C) Phenotypes of seedlings transferred from MS to MS+NaCl (100 mM) medium. The photograph was taken 5 days after 3-day-old seedlings were transferred. (D) Survival rate of the wild type, *lrx345* and *aba2-1 lrx345* seedlings grown on MS+NaCl (120 mM) media supplemented with or without ABA (1 μM). (E) ABA contents of 10-day-old seedlings before and after NaCl (150 mM) treatment (1 h). (F) qRT-PCR analysis of the transcript levels of *PDF1.2*, *PDF1.3*, *PR1* and *PR5* in the indicated genotypes. *ACTIN8* was used as an internal control. Values in (B–F) are means of three biological replicates ± SD, *P < 0.05 and **P < 0.01 (Student's *t*-test). (G) Measurement of JA and SA contents in the wild type, *lrx345* and *aba2-1 lrx345* seedlings. Values are means of three biological replicates ± SD. Different letters represent statistically significant differences (*P* < 0.01, one-way ANOVA). (H) qRT-PCR analysis of the expression of *RD29A* and *RD29B* in the indicated genotypes before and after NaCl treatment. *ACTIN8* was used as an internal control. Values are means of three biological replicates ± SD.

As many salt stress-responsive genes were down-regulated in the *lrx345* and *fer-4* mutants after NaCl treatment for 6 h, we speculated that the salt-hypersensitivity of *lrx345* and *fer-4* mutants may be caused by an impaired stress response. ABA is an important hormone for the up-regulation of stress-responsive genes in plants [32]. To determine whether the salt-hypersensitivity of the *lrx345* mutant is associated with a defective ABA pathway, we crossed the *lrx345* mutant with the *aba2-1* mutant, in which ABA biosynthesis is substantially decreased [33] (Fig. S6A). Unexpectedly, the *aba2-1* mutation almost fully rescued the cell-death phenotype of the *lrx345* mutant under high salinity (Fig. 4A–C). To confirm this result, we crossed the *lrx345* mutant with another *aba2* mutant allele, *aba2-3*. Similarly, the *aba2-3* mutation almost fully suppressed the salt-hypersensitivity of the *lrx345* mutant (Fig. S6B and C). The suppression of the cell-death phenotype of the *lrx345* mutant by *aba2* mutation under high salinity was alleviated by applying exogenous ABA (Fig. 4D). The salt-hypersensitivity of the *fer-4* mutant was also suppressed by the *aba2-1* mutation (Fig. S6D and E). We then tested the accumulation of ABA in the *lrx345* and *fer-4* mutants and found that ABA content was higher in these mutants than in the wild type plants, both in basal conditions and upon salt treatment (Fig. 4E). Gene expression analysis showed that ABA *de novo* biosynthesis genes were not significantly up-regulated and that ABA catabolism genes were not significantly down-regulated in the *lrx345* mutant (Table S4). However, the β-glucosidase gene *AtBG1*, which is required for conversion of the inactive glucose-conjugated form of ABA (ABA-GE) to the active form of ABA [34], was highly up-regulated in the *lrx345* mutant (Table S4), suggesting that a LRX3/4/5-mediated pathway may modulate ABA accumulation via the regulation of *AtBG1* expression.

We found that the *aba2-1* single mutant exhibited a higher survival rate than the wild type when grown on the medium with a high concentration of NaCl (150 mM) (Fig. S6F and G), suggesting that accumulation of ABA promotes salt-induced cell death. Consistent with the previous study showing that *fer* mutant plants are hypersensitive to ABA [17,35], the *lrx345* mutant also exhibited delayed seed germination on the medium supplemented with ABA (Fig. S6H).

Previous study showed that the increased sensitivity of *fer* mutant plants to ABA may be a result of inactivation of ABI2 [17,35], a phosphatase that negatively regulates ABA signaling. To test whether the hypersensitivity of *lrx345* and *fer-4* to salt stress involves ABI2, we crossed *lrx345* and *fer-4* mutants with the *abi2-1* mutant. The *abi2-1* is a dominant mutation that blocks ABA signaling [36]. We found that the *abi2-1* mutation could not suppress the salt-hypersensitive phenotype of the *lrx345* and *fer-4* mutants (Fig. S7), suggesting that ABI2 alone cannot account for LRX3/4/5- and FER-mediated regulation of salt stress responses.

The *aba2-1* mutation also partially suppressed the growth defects and anthocyanin accumulation of the *lrx345* mutant (Fig. S6I–K). The transcript levels of *PDF1.2* and *PDF1.3* were markedly lower, while the transcript levels of *PR1* and *PR5* were moderately lower in the *aba2-1 lrx345* quadruple mutant than that in the *lrx345* mutant (Fig. 4F), which is consistent with a drastic reduction in JA content and a slight decrease in SA content in the quadruple mutant (Fig. 4G). These results indicate that ABA accumulation contributes to the activation of JA signaling pathway and partially contributes to the activation of SA signaling pathway in the *lrx345* mutant.

### LRX345-FER module is required for both activation and attenuation of salt stress-responsive genes

**Figure 5.**
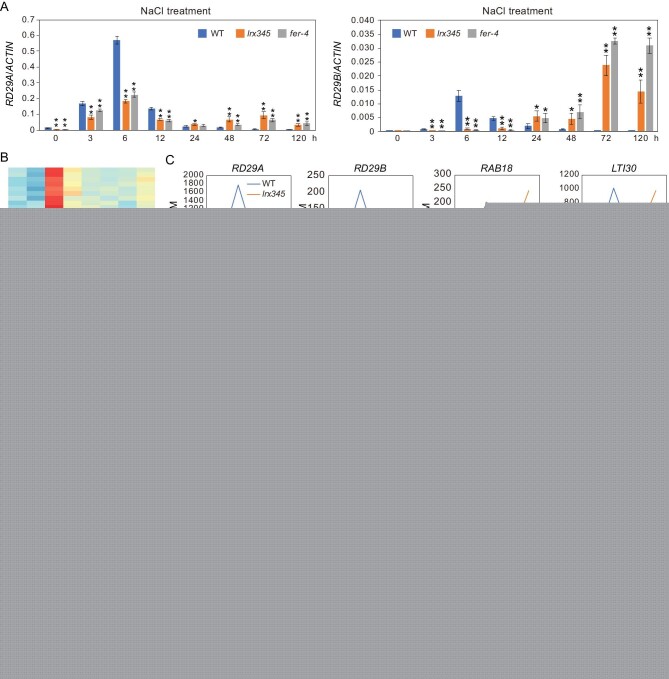
LRX3/4/5 and FER are required for the dynamic regulation of salt stress-responsive gene expression. (A) Ten-day-old seedlings of the wild type, *lrx345* and *fer*-4 were treated with NaCl for 0, 3, 6, 12, 24, 48, 72 and 120 h. The transcript levels of *RD29A* and *RD29B* were analyzed by qRT-PCR. *ACTIN8* was used as an internal control. (B) RNA-seq assay was performed for the wild type and *lrx345* after NaCl treatment for 0, 6, 24 and 72 h. Heat map shows the genes that exhibited lower transcript levels after NaCl treatment for 6 h, and showed higher transcript levels after treatment for 72 h in the *lrx345* mutant compared with that in the wild type. (C) Representative genes that display similar expression patterns as *RD29A* and *RD29B* after salt treatment for 0, 6, 24 and 72 h. The transcript profile of each gene in the wild type and *lrx345* mutant was generated from RNA-seq data. (D) Wild type seedlings were pretreated with or without EGCG (100 μM) for 2 h before being subjected to NaCl treatment for 0, 6, 24 and 72 h. Transcript levels of *RD29A*, *RD29B* and *RAB18* were analyzed. *ACTIN8* was used as an internal control. Values in (A) and (D) are means of three biological replicates ± SD, *P < 0.05 and **P < 0.01 (Student's *t*-test).

Although the *aba2-1* mutation suppressed the cell-death phenotype of the *lrx345* mutant under high salinity, the salt-induced expression of *RD29A* and *RD29B* was further impaired in the *aba2-1 lrx345* quadruple mutant (Fig. 4H), suggesting that the cell-death phenotype of the *lrx345* triple mutant under high salinity is not caused by the decreased expression of these salt stress-responsive genes. It has been proposed that the outcome of salt stress responses (adaptation or cell death) depends to some extent on when exactly these responses start and end [37]. Overactivation or prolonged activation of stress responses is apparently detrimental for plants under stress conditions. To test whether LRX3/4/5 and FER may be involved in the attenuation of salt stress-responsive genes, we examined the expression of *RD29A* and *RD29B* genes after exposure to high salinity for 0, 3, 6, 12, 24, 48, 72 and 120 h. In the wild type, the expression of both *RD29A* and *RD29B* reached peak levels after salt treatment for 6 h, and then went down after longer exposure to high salinity (Fig. 5A). In the *lrx345* and *fer-4* mutants, the expression of *RD29A* also reached peak levels after salt treatment for 6 h but showed a second smaller wave of expression after 24 h, while the expression of *RD29B* continuously increased after exposure to salt stress (Fig. 5A). Similarly, the expression of *RD29A* and *RD29B* genes was increased in both *RALF22* and *RALF23* overexpressing plants compared with the wild type after prolonged exposure to high salinity (Fig. S8A). These results indicate that the LRXs-RALFs-FER module is not only required for the up-regulation of salt stress-responsive genes soon after salt treatment, but is also essential for attenuating the expression of salt stress-responsive genes after longer exposure to salt stress. The increased expression of *RD29A* and *RD29B* genes in the *lrx345* mutant after salt treatment for 72 h was completely abolished by the *aba2* mutation (Fig. S8B), suggesting that the ABA-mediated pathway is required for prolonged expression of salt stress-responsive genes in the *lrx345* mutant.

To investigate how many salt stress-responsive genes display prolonged expression in the *lrx345* mutant under salt stress, we performed RNA-seq analysis for the wild type and *lrx345* mutant after salt treatment for 0, 6, 24 and 72 h (Table S5). RNA-seq data confirmed that the expression of *RD29A* and *RD29B* genes was lower at 6 h but higher at 72 h after NaCl treatment in the *lrx345* mutant than in the wild type (Fig. 5B and C, Table S6). We identified 160 salt stress-inducible genes, including *RAB18*, *LTI30*, *ABR*, *DAA1*, *NADP-ME1*, *TZF4*, *ABCG6*, *AT1G16850* and *LEA4-5*, that displayed similar expression patterns to *RD29A* and *RD29B* (Fig. 5B and C, Table S6). The abnormal expression patterns of these genes in the *lrx345* mutant were confirmed by qRT-PCR analysis (Fig. S9). Gene ontology (GO) enrichment analysis of these 160 genes showed that they are enriched in the categories ‘phenylpropanoid metabolic process’ (*P* value = 1.19E-12), ‘response to acid chemical’ (*P* value = 5.17E-10), and ‘response to oxygen-containing compound’ (*P* value = 1.54E-08). However, 173 salt stress-inducible genes, including *COR15B*, *COR47*, *COR413-TM1*, *KIN2* and *RD22*, had lower expression in the *lrx345* mutant than in the wild type after salt treatment for 6 h but did not show higher expression in the mutant after salt treatment for 72 h (Table S7). These genes are enriched in the categories ‘starch metabolic process’ (*P* value = 3.35E-06), ‘lipid metabolic process’ (*P* value = 4.72E-06) and ‘glucan metabolic process’ (*P* value = 1.28E-05). Together, these results suggest that the LRX3/4/5- and FER-mediated pathway is required for both activation and attenuation of a specific set of stress-responsive genes.

LRXs and FER maintain cell wall integrity likely via a communication with pectin [18,38]. To understand whether the abnormal expression pattern of stress-responsive genes in the *lrx345* and *fer-4* mutants results from disrupted cell wall properties under high salinity, we pretreated wild type plants with epigallocatechin gallate (EGCG), an inhibitor of pectin methylesterases (PMEs) [39], before subjecting them to salt treatment for 0, 6, 24 and 72 h. PMEs are enzymes that remove methyl ester groups from homogalacturonan (HGA), a major constituent of pectin in the cell wall [40]. Demethylesterified pectins either form Ca^2+^ bonds and contribute to wall firmness or are more accessible to pectin-degrading enzymes [41]. Gene expression analysis showed that, similar to the *lrx345* and *fer-4* mutants, the wild type seedlings pretreated with EGCG lost the ability to maintain proper expression patterns of *RD29A*, *RD29B* and *RAB18* genes after both short and prolonged exposure to high salinity conditions (Fig. 5D). These results suggests that EGCG-triggered modifications of cell wall components likely determine the expression patterns of stress-responsive genes under high salinity. Consistent with a previous study [38], we found that EGCG treatment inhibited the root elongation of the wild type plants, but had a less effect on the root growth inhibition of *fer-4* mutant (Fig. S10).

### Accumulation of reactive oxygen species (ROS) is responsible for salt-induced cell death of the *lrx345* and *fer-4* mutants

**Figure 6.**
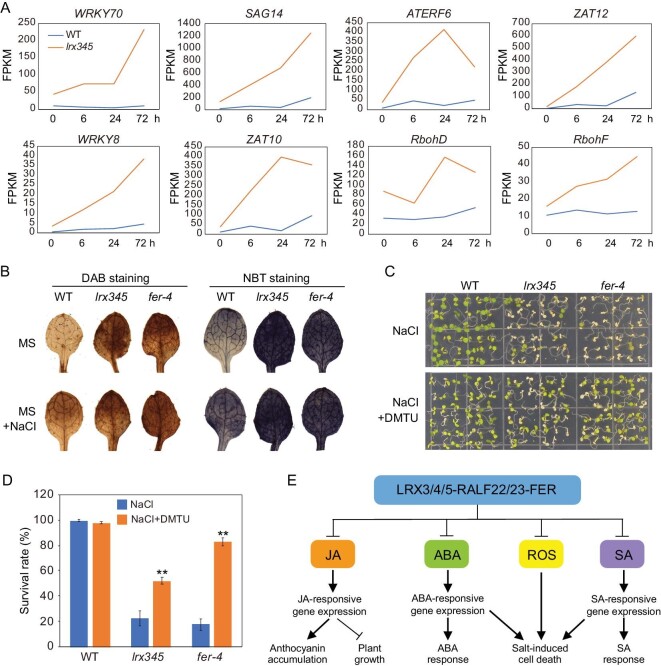
Inhibition of ROS production restores the cell-death phenotype of *lrx345* and *fer-4* mutants under high salinity. (A) Transcript levels of *RbohD*, *RbohF* and six oxidative stress-responsive genes before and after NaCl treatment. The expression values of each gene were calculated based on RNA-seq data. (B) Wild type, *lrx345* and *fer-4* seedlings before and after NaCl treatment were stained with DAB and NBT. (C) Phenotype of seedlings grown on MS+NaCl (120 mM) media supplemented with or without DMTU (5 mM). (D) Survival rate of the wild type, *lrx345* and *fer-4* seedlings grown on MS+NaCl (120 mM) media supplemented with or without DMTU (5 mM). Values are means of three biological replicates ± SD, **P < 0.01 (Student's *t*-test). (E) Diagram summarizing the role of LRX3/4/5-RALF22/23-FER module in the regulation of JA, SA, ABA and ROS accumulation.

Our RNA-seq data also revealed that genes constitutively up-regulated in the *lrx345* mutant were enriched in the category ‘response to oxidative stress’ (*P* value = 1.97E-07). Specifically, 59 oxidative stress-responsive genes, including *WRKY8*, *WRKY70*, *SAG14*, *ATERF6*, *ZAT10* and *ZAT12*, were constitutively up-regulated in the *lrx345* mutant (Fig. 6A). Under high salinity, the transcripts of these six genes either did not change or slowly increased in the wild type, but they were dramatically up-regulated in the *lrx345* mutant (Fig. 6A). Interestingly, *RbohD* and *RbohF*, encoding two NADPH oxidative enzymes required for the production of ROS, were up-regulated in the *lrx345* mutants both under basal conditions and upon NaCl treatment (Fig. 6A). In addition, the protein level of RbohD was increased in the *lrx345* mutant under both normal conditions and salt stress treatment (Fig. S11A).

We measured the content of ROS, including hydrogen peroxide (H_2_O_2_) and superoxide radicals (O_2_^·−^), in the *lrx345* and *fer-4* mutants using 3,3′-diaminobenzidine (DAB) and nitro blue tetrazolium (NBT) staining. Levels of both H_2_O_2_ and O_2_^·−^ were higher in the leaves of the *lrx345* and *fer-4* mutants than in wild type leaves (Fig. 6B). The increased accumulation of ROS in the leaves of the *lrx345* and *fer-4* mutants is opposite to previous results showing that FER promotes the accumulation of ROS in the root and filiform apparatus and positively regulates flg22-, elf18- and chitin-induced ROS production [14,42,43]. These contradicting results suggest that FER may control ROS production in tissue-specific and environmental stress-dependent manners. To understand whether the salt-induced cell-death phenotype of the *lrx345* and *fer-4* mutants is caused by the increased production of ROS, we grew seedlings on NaCl medium supplemented with dimethylthiourea (DMTU), a scavenger of H_2_O_2_. Application of DMTU largely reversed the cell-death phenotype of *fer-4* mutant and to a lesser extent suppressed that of *lrx345* mutant under high salinity (Fig. 6C and D). We also found that the wild type plants grown on NaCl media supplemented with H_2_O_2_ displayed a lower survival rate than the plants grown on media with NaCl alone (Fig. S11B and C), supporting that ROS over-accumulation enhances salt-induced cell death in plants.

Study has shown that ABA promotes the production of ROS via RbohD and RbohF NADPH oxidases [44]. To investigate whether *aba2* mutation-mediated inhibition of the cell-death phenotype of *lrx345* and *fer-4* mutants is a result of reduced ROS production, we assessed H_2_O_2_ and O_2_^·−^ in the *aba2-1 lrx345* and *aba2-1 fer-4* mutants using DAB and NBT staining. The *aba2* mutation did not obviously reduce the accumulation of H_2_O_2_ and O_2_^·−^ in the *lrx345* and *fer-4* mutants (Fig. S12A). However, gene expression analysis indicated that *RbohF*, but not *RbohD*, was substantially down-regulated in the *aba2-1 lrx345* mutant compared with the *lrx345* mutant (Fig. S12B). In addition, the expression of ROS-responsive genes, such as *ZAT10*, *SAG1*, *WRKY8* and *AtERF6*, was largely attenuated in the *aba2-1 lrx345* mutant, especially after NaCl treatment for 24 h or longer (Fig. S12B). These results suggest that the suppression of the salt-induced cell death of the *lrx345* and *fer-4* mutants by *aba2* mutation is at least partially a result of attenuated ROS response under salt stress.

## DISCUSSION

Environmental stresses, such as high salinity, not only trigger intracellular stress responses, but also interfere with cell wall integrity and inhibit plant growth [45]. Coordinated regulation of cell wall integrity, growth and stress response is critical for plants to survive under unfavorable environmental conditions. Previous results have indicated that the LRX3/4/5-RALF22/23-FER module is involved in regulating cell wall integrity, growth and salt stress responses in plants. Here, we provide transcriptomic, hormonal and genetic data to show that this regulation is achieved by the modulation of JA, SA and ABA hormones (Fig. 6E). As several genes involved in JA and SA biosynthesis were constitutively up-regulated in the *lrx345* and *fer-4* mutants, we proposed that LRX3/4/5-RALF22/23-FER may regulate JA and SA pathways by controlling the expression of their biosynthesis genes. A recent study showed that SA- and JA-responsive genes are also highly up-regulated in the *fer-4* mutant and that FER interacts with and destabilizes MYC2, a master regulator of JA response [19]. These results suggest that FER may regulate the expression of JA biosynthesis genes via the modulation of MYC2 stability.

Genetic or chemical perturbation of the cell wall was known to activate JA signaling [46,47], but the components linking cell wall stress to JA signaling had been missing. We found that the JA pathway is constitutively activated in the *lrx345* and *fer-4* mutants and in transgenic plants overexpressing *RALF22* or *RALF23*, suggesting that the LRX3/4/5-RALF22/23-FER module is involved in the relay of cell wall stress signals to the JA pathway. It is likely that stress-induced perturbation of cell wall integrity triggers the dissociation of the RALF22/23 from LRX3/4/5, and the released RALF22/23 peptides promote the internalization of FER [12], which then triggers the activation of the JA pathway. Our results showed that the SA pathway, which is generally considered as antagonistic to the JA signaling pathway [48,49], is also constitutively up-regulated in the *lrx345* and *fer-4* mutants. Therefore, how these two hormones are simultaneously increased in the *lrx345* and *fer-4* mutants is of great interest. Although disruption of SA biosynthesis partially suppresses the salt-hypersensitivity of the *lrx345* mutant, further work is required to address whether SA is induced by the perturbation of cell wall integrity and the exact roles of SA in the LRX3/4/5-RALF22/23-FER module-mediated pathways.

JA is an important hormone that is required for defense against diverse pathogens and herbivores [50,51]. The JA-mediated defense responses are usually accompanied by growth repression. In recent years, the molecular mechanisms underlying the roles of JA in the regulation of plant growth have been well addressed. JA promotes the accumulation of DELLA proteins that interact with and repress the transcription activity of growth-promoting PIF transcription factors [52–54]. Our genetic analysis showed that the dwarf phenotype of the *lrx345* and *fer-4* mutants is almost fully suppressed by mutations in *COI1* and *AOS* genes, which shows that JA is involved in the regulation of plant growth in the cell wall integrity pathways. Studies have shown that salt stress can activate the JA pathway [55,56], but the biological significance of JA pathway in salt stress tolerance is still largely unknown. We propose that the JA pathway, which is triggered by salt-induced perturbation of cell wall integrity via the LRX3/4/5-RALF22/23-FER module, is required to inhibit cell expansion under high salinity. The inhibition of cell expansion under high salinity allows priority to cell wall repair before cell growth can resume, thus preventing cell bursting.

The enhanced cell death of the *lrx345* and *fer-4* mutants under high salinity is similar to that observed in the *sos1* mutant [57]. SOS1, a Na^+^/H^+^ antiporter, is required for the extrusion of excessive Na^+^ from the cytosol and maintainance of ion homeostasis under salt stress [57,58]. Unlike the *sos1* mutant, Na^+^ is not significantly accumulated in the *fer* mutant under salt stress [59], which suggests a different mechanism for the salt-induced cell death in the *lrx345* and *fer* mutants. Salt stress can induce diverse cellular responses, such as Ca^2+^, ROS and ABA [32]. Our results showed that ABA and ROS contents are consitutively elevated in the *lrx345* and *fer-4* mutants, and the cell-death phenotype of the *lrx345* and *fer-4* mutants is almost fully suppressed by the *ABA2* mutation that disrupts ABA biosynthesis, and largely suppressed by DMTU, a H_2_O_2_ scavenger, suggesting that the abnormal ABA homeostasis and accumulation of ROS are the causes of the cell-death phenotype of the *lrx345* and *fer-4* mutants under high salinity. The accumulation of ROS in the *lrx345* and *fer-4* mutants is probably caused by the increased expression of *RbohD* and *RbohF* genes. Extensive studies have shown that over-accumulation of ROS results in cell death in plants [60–62], but the mechanisms underlying the roles of ABA in the regulation of cell death are still largely unknown. Based on the knowledge that ABA promotes the production of ROS via RbohD and RbohF NADPH oxidases [44], and that elevated expression of *RbohF* gene in the *lrx345* mutant was attenuated by the *aba2* mutation, we speculate that ABA promotes the salt-induced cell death of the *lrx345* and *fer-4* mutants via RbohF-mediated production of ROS. We found that the *abi2-1* mutation did not suppress the salt-hypersensitive phenotype of *lrx345* and *fer-4* mutants. This result may be explained because the *abi2-1* mutation does not fully block ABA signaling. Alternatively, LRX3/4/5- and FER-mediated regulation of salt stress response may depend on ABA pathway component(s) other than ABI2. Studies have shown that FER regulates the GEFs-ROP11 complex [17] and ROP11 protects the activity of ABI1 and ABI2 by alleviating the ABA receptor PYL9-mediated inhibition [63]. It is possible that besides regulating ABA accumulation, FER may control salt stress tolerance by regulating ABA response through several signaling components.

Proper regulation of stress-responsive gene expression is critical for plants to survive under adverse environmental conditions. Although extensive studies have elucidated the genes and signaling pathways that are required for the regulation of the expression of stress-responsive genes [32,64,65], the physical properties of cells that determine the dynamic expression of stress-responsive genes in response to high salinity are still unclear. Our data showed that the *lrx345* and *fer-4* mutants, and the wild type plants treated with EGCG, an inhibitor of PMEs, lost the ability to activate and attenuate the expression of many stress-responsive genes after short and prolonged exposure to high salinity, suggesting that expression patterns of salt stress-responsive genes are determined by the properties of the cell wall, the signals of which are monitored and transduced by the LRX3/4/5-RALF22/23-FER module. In future, the association between pectin structure and salt stress response needs to be investigated in more detail.

In summary, identification of the downstream signaling pathways that are regulated by the LRXs-RALFs-FER module helps us to understand the intracellular events that plants explore to respond to cell wall perturbation under a variety of environmental stresses. The intermediate steps leading from the LRX3/4/5-RALF22/23-FER module to the induction of downstream responses including the increases in ABA, JA and SA levels remain to be elucidated. Nonetheless, our results provide a mechanistic framework to understand how phytohormone content and signaling are modulated to provide a homeostatic mechanism allowing for maintenance of cell wall integrity and growth regulation as well as for plant survival under salt stress. The simultaneous increase of multiple hormones in the *lrx345* and *fer-4* mutants provides an invaluable opportunity to investigate the cross-talk among these hormones and how too much of these defense hormones under salt stress can cause plant death. Understanding the biological function of the LRX3/4/5-RALF22/23-FER module may ultimately enable engineering of crop plants that not only can survive salt stress but also can grow well.

## METHODS AND MATERIALS

The methods and materials are described in detail in the Supplementary data.

## Data resources

RNA-seq data have been deposited in the NCBI GEO under accession numbers GEO: GSE136269.

## Supplementary Material

nwaa149_Supplemental_Files
