# Supplementary material for: The LRXs-RALFs-FER module controls plant growth and salt stress responses by modulating multiple plant hormones
Source: Natl Sci Rev. 2020 Jun 30;8(1):nwaa149. doi: 10.1093/nsr/nwaa149 (PMC8288382; doi:10.1093/nsr/nwaa149)
Supplement: nwaa149_Supplemental_Files [file nwaa149_supplemental_files.zip › METHODS AND MATERIALS.docx]

**METHODS AND MATERIALS**

**Plant materials and growth conditions**

The Col-0 ecotype of *Arabidopsis thaliana* was used as the wild type. *lrx345*, *fer-4*, *coi1-1*, *aos*, *coi1-16*, *aba2-1*, *aba2-3*, *abi2-1*, *sid2-2*, and *jazQ* mutants have been described previously [12,24,25,27,28,33,36,66,67]. The *coi1-1 lrx345*, *aos lrx345*, *coi1-16 lrx345*, *aba2-1 lrx345*, *aba2-3 lrx345*, *sid2-2 lrx345*, *coi1-1 fer-4*, and *aba2-1 fer-4* mutants were generated by crossing. Homozygous mutants were confirmed by PCR-based genotyping. Plants were grown at 23°C with a long-day light cycle (16 h light/8 h dark). The primers used for genotyping are listed in Table S8.

**Mapping by whole-genome resequencing**

To screen for *lrx345* suppressors, approximately 15,000 *lrx345* seeds were mutagenized by EMS. In M2 generation, suppressors that recovered the growth of *lrx345* mutants were collected for mapping. The *lrx345* suppressors were backcrossed to the *lrx345* mutant. In the F2 generation, approximately 120 plants with suppressor phenotypes were mixed together for the extraction of genomic DNA using DNeasy Plant Maxi kit (QIAGEN). Illumina technology was used for whole genomic DNA re-sequencing. The sequencing reads generated from *lrx345* suppressors were aligned to the TAIR10 reference genome using BWA [68]. The generated SAM files were converted to BAM files. PCR duplications were removed using MarkDuplicates.jar in Picard (http://broadinstitute.github.io/picard/). SNPs in the suppressors were identified using HaplotypeCaller in GATK [69]. The SNPs were chosen based on the following criteria: 1) homozygous reference alleles in wild type; 2) C->T or G->A mutation change in the suppressors; 3) ratios between alternative alleles and total read depth > 0.3. After identifying all SNPs, a five-SNP window was used to calculate the ratio of mutations. The position with high mutation ratio was marked on the chromosome. The SNPs around the peak position were selected to identify the causal mutations.

**Construction of plasmids and transformation**

To generate *p35S::JAZ1-YFP* and *p35S::JAZ9-YFP* constructs, the coding region sequences of *JAZ1* and *JAZ9* were amplified and integrated into pDONR207 ENTRY vector using Gateway BP Clonase II Enzyme Mix (Thermo Fisher Scientific). After verification by sequencing, all fragments were recombined into the destination vector pEarleyGate101 using Gateway LR Clonase II Enzyme Mix (Thermo Fisher Scientific). Genetic transformation of *Arabidopsis thaliana* was conducted by using *Agrobacterium tumefaciens* (GV3101 strain)-mediated floral dip method. The homozygous lines of each transformant were screened on MS medium supplemented with phosphinothricin. Primers used for the construction are listed in Table S8.

**Protein Extraction and Immunoblotting**

Proteins were extracted from 7-day-old seedlings of *Arabidopsis thaliana* using the following buffer: 100 mM Tris-HCl pH7.5, 150 mM NaCl, 10% Glycerol, 10 µg/mL antipain, 10 µg/mL aprotinin, 10 µg/mL leupeptin, 1 mM DTT, 1 mM PMSF and phosphatase inhibitor cocktail set II. The ground samples were mixed with the extraction buffer by vortex and incubated on ice for 15 min. After centrifugation at 4 °C for 10 min at a maximum speed, the supernatants were transferred into new 1.5 mL eppendorf tubes. The total protein concentration was measured by BioPhotometer (Eppendorf) using Quick Start^TM^ Bradford 1×Dye Reagent (BIO-RAD). An equal amount of protein for each sample was loaded and separated by sodium dodecyl sulfate polyacrylamide gel electrophoresis (SDS-PAGE). Proteins on the gel were transferred to supported nitrocellulose membrane (BIO-RAD) using a trans-blot semi-dry transfer cell (BIO-RAD). Immunoblotting was performed by using anti-GFP.

**DAB and NBT staining**

DAB staining solution (DAB was dissolved in 50 mM Tris-HCl pH 3.0 with a final concentration of 1 mg/mL) and NBT staining solution (NBT was dissolved in water with a final concentration of 1 mg/mL) were used for hydrogen peroxide and superoxide detection, respectively. For the staining assay, the seeds of *Arabidopsis thaliana* were germinated vertically on 1/2 MS-agar plates for 10 days, and then the seedlings were transferred into liquid 1/2 MS medium and incubated overnignt with a gentle shake. After treatment with 150 mM NaCl for 1 h, the seedlings were stained with DAB and NBT staining solution and incubated overnight at room temperature under dark conditions. Finally, the seedlings were destained overnight by ethanol and visualized by LEICA EZ4 HD microscope.

**Measurement of ABA, JA, and SA hormones**

For ABA analysis, approximately 50 mg fresh seedlings were ground in liquid nitrogen and placed in a 1.5 mL Eppendorf tube with 0.5 mL 70% MeOH containing 2 ng ABA-*d_6_*. For JA analysis, 50 mg ground seedlings were placed in a 1.5 mL tube with 0.5 mL 70% MeOH. For SA analysis, 25 mg ground plant samples were placed in a 1.5 mL tube with 0.5 mL 70% MeOH containing 2 ng SA-*d*_4_. To measure these three hormones, the tubes wgere vortexed at 100 *g* for 1 h at 10°C, and then centrifuged at 20,000 *g* for 10 min at 20°C. 0.3 mL supernatant from each tube was transferred to a new HPLC vial and diluted two-fold with sterile distilled water. 50 µL solution of each sample was assayed by liquid chromatography tandem mass spectrometry (LC-MS) and the levels of ABA, JA, and SA were quantified by Peakview 1.2 (AB SCIEX). JA concentration was calculated based on the calibration curve created by running a JA standard solution.

**Anthocyanin measurement**

Anthocyanin content was assessed as previously described [70]. In brief, 30 seedlings for each sample were collected in a 2 mL Eppendorf tube with 600 μL of 1% HCl in methanol (v/v). The seedlings were incubated overnight in the dark at 4°C with gentle shaking. After extraction, 400 μL water and 400 μL chloroform were added and the samples were vortexed. The tubes were centrifuged at 12,000 rpm for 2 min, and the supernatant was transferred to a new tube. The absorbance of each supernatant was measured spectrophotometrically at 530 nm and 657 nm, respectively. The concentration of anthocyanin was calculated using the formula *A*_530_ - 0.25 *A*_657_.

**RNA-seq analysis**

Ten-day-old seedlings before and after NaCl treatment (150 mM) for 6 h were collected for RNA isolation using Trizol reagent (Invitrogen) according to the manufacturer’s instructions. The total RNAs were sequenced by Illumina. Three biological replicates were performed for each sample. Reads of RNA-seq data were mapped to the Arabidopsis reference genome (TAIR10) using TopHat [71]. Differentially expressed genes between wild type and *lrx345* mutant were identified using cuffdiff in Cufflinks. The genes belonging to the categories of “JA biosynthetic genes”, “SA biosynthetic genes”, “response to JA”, and “response to SA” were identified based on the annotation in the database of The Arabidopsis Information Resource (TAIR).

**Quantitative real-time RT-PCR Analysis**

Ten-day-old seedlings before and after NaCl or ABA treatment were collected for RNA isolation using plant RNA kit (Omega Bio-tek) according to the manufacturer’s instructions. Complementary DNA was synthesized from 2 μg total RNA using M-MLV reverse transcriptase (Promega). Quantitative real-time PCR was performed with PerfeCTa SYBR Green Fastmix (Quanta Biosciences). Primers used for qRT-PCR are shown in Table S8.
