# Supplementary material for: The LRXs-RALFs-FER module controls plant growth and salt stress responses by modulating multiple plant hormones
Source: Natl Sci Rev. 2020 Jun 30;8(1):nwaa149. doi: 10.1093/nsr/nwaa149 (PMC8288382; doi:10.1093/nsr/nwaa149)

Figure S5

A

Suppresors	Aelles	Mutation	aa changes
slrx4	*tt4-16*	C284 to T	Ser95 to Phe
slrx9	*tt4-17*	G332 to A	Gly111 to Asp
slrx26	*tt4-18*	C323 to T	Pro108 to Leu
slrx398	*tt4-19*	C574 to T	Leu192 to Phe
slrx720	*tt4-20*	C833 to T	Pro278 to Leu
slrx429	*tt3-7*	G317 to A	Gly106 to Glu
slrx621	*tt6-7*	G331 to A	Gly111 to Arg
slrx748	*tt6-8*	G201 to A	Trp67 to stop

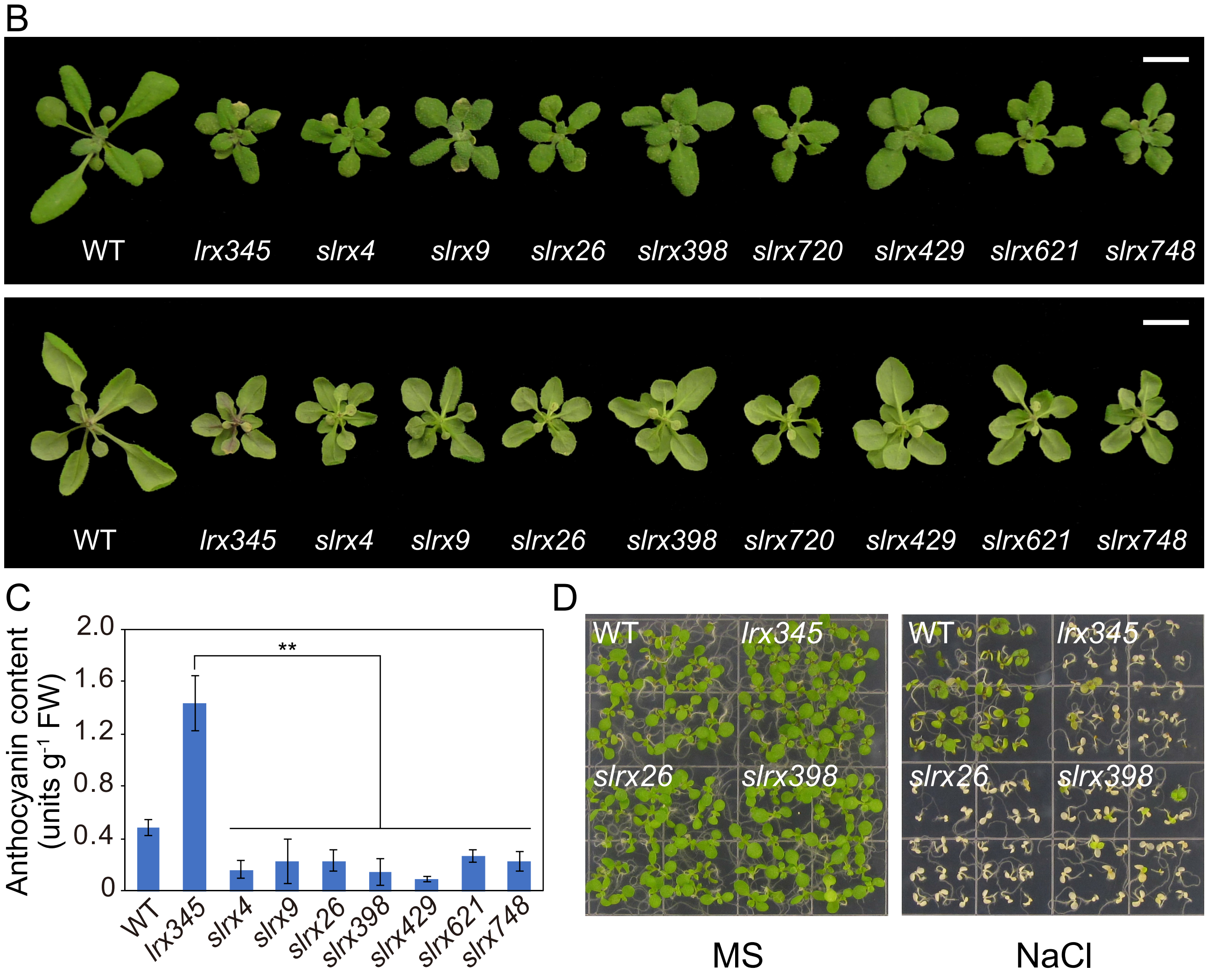

Supplement: nwaa149_Supplemental_Files [file nwaa149_supplemental_files.zip › SFigure 5.pdf]
