# Supplementary material for: The LRXs-RALFs-FER module controls plant growth and salt stress responses by modulating multiple plant hormones
Source: Natl Sci Rev. 2020 Jun 30;8(1):nwaa149. doi: 10.1093/nsr/nwaa149 (PMC8288382; doi:10.1093/nsr/nwaa149)
Supplement: nwaa149_Supplemental_Files [file nwaa149_supplemental_files.zip › Supplementary Table 8.docx]

Table S8. Primers used in this study.

| Primer name | Sequence | Usage |
| --- | --- | --- |
| PDF1.2 RT-LP | GCTTCCATCATCACCCTTATCTT | Quantitative real-time PCR |
| PDF1.2 RT-RP | TAGTTGCATGATCCATGTTTGG | Quantitative real-time PCR |
| PDF1.3 RT-LP | CTAAGTCTGCTGCCATCATCACT | Quantitative real-time PCR |
| PDF1.3 RT-RP | ATGTTTTGCCCCCTCAAGGT | Quantitative real-time PCR |
| PR1 RT-LP | TTAGCCTGGGGTAGCGGTGACT | Quantitative real-time PCR |
| PR1 RT-RP | GGCACATCCGAGTCTCACTGAC | Quantitative real-time PCR |
| PR5 RT-LP | GCCCCGACATGCTTAAGGTCAT | Quantitative real-time PCR |
| PR5 RT-RP | GCGTTCTTGAAAATCCTCGAGT | Quantitative real-time PCR |
| RD29A RT-LP | CTTGTCGACGAGAAGCAAAGAA | Quantitative real-time PCR |
| RD29A RT-RP | TCTTGATGGAGAATTCGTGTCC | Quantitative real-time PCR |
| RD29B RT-LP | GAGAAGGGAGCATCCAAAGTGT | Quantitative real-time PCR |
| RD29B RT-RP | ATACACTGGTGCGCCGTGAACT​ | Quantitative real-time PCR |
| AOS RT-LP | GCTTTTCGATTCTTTGGAGAAA | Quantitative real-time PCR |
| AOS RT-RP | CTCGGTAAACCAATAGAGAGTAATG | Quantitative real-time PCR |
| LOX3 RT-LP | GTCGTAGTTGATACCCTCCAAGA | Quantitative real-time PCR |
| LOX3 RT-RP | GTTGTGGACCGTAGATCTTGGG | Quantitative real-time PCR |
| AOC2 RT-LP | CAAAACGGGAATATCGAAAACC | Quantitative real-time PCR |
| AOC2 RT-RP | TTCTCTGGGACGTGTTCGATGA | Quantitative real-time PCR |
| LOX4 RT-LP | AAGTCGGTGAAGTTTAAGGTGA | Quantitative real-time PCR |
| LOX4 RT-RP | TCTTCTTTGGCTCATTCGTTTT | Quantitative real-time PCR |
| AOC3 RT-LP | CTACTACTTCCAAGAACCTCAACG | Quantitative real-time PCR |
| AOC3 RT-RP | TGGTTTCTTGCCGAGTTTAAGA | Quantitative real-time PCR |
| OPCL1 RT-LP | TCCGAGATAGGGATTCGCAAAG | Quantitative real-time PCR |
| OPCL1 RT-RP | GGACTGGATTTGAATCCTTAATCTG | Quantitative real-time PCR |
| RbohD RT-LP | TCCACGCACTCAAAGGTCTC | Quantitative real-time PCR |
| RbohD RT-RP | GACGTTATTCCGGCGAGCTA | Quantitative real-time PCR |
| RbohF RT-LP | TGACACGCCAAGACGAAAGA | Quantitative real-time PCR |
| RbohF RT-RP | GAGCAGAACGAGCATCACCT | Quantitative real-time PCR |
| ZAT12 RT-LP | GACACAGGAACGAGAGTGGG | Quantitative real-time PCR |
| ZAT12 RT-RP | TTCAACGTAGTCACCGTGGG | Quantitative real-time PCR |
| SAG14 RT-LP | GAAAGAGAAACCCATTAGCCAC | Quantitative real-time PCR |
| SAG14 RT-RP | GTACTTGGGGTTGATCCAGGTG | Quantitative real-time PCR |
| WRKY8 RT-LP | CGTCGGACAGCAATGTTCTCTG | Quantitative real-time PCR |
| WRKY8 RT-RP | CTCACTCTCCTGTTGATGAAATCC | Quantitative real-time PCR |
| ATEFR6 RT-LP | CTCCTCGCGTTACTGTTCAATC | Quantitative real-time PCR |
| ATEFR6 RT-RP | AACACGAGTTCCACGACGAGTC | Quantitative real-time PCR |
| JAZ1 RT-LP | GCCAATCCAATCCTCCCCAA | Quantitative real-time PCR |
| JAZ1 RT-RP | TGCCTGTGGTTTGAGGGTTT | Quantitative real-time PCR |
| XTH14 RT-LP | GGTAAAGGCGACCGTGAGAT | Quantitative real-time PCR |
| XTH14 RT-RP | AGCCACCCCATTTTTCTCGT | Quantitative real-time PCR |
| AT1G05660 RT-LP | GTCACGGGGTTAGCATAGGG | Quantitative real-time PCR |
| AT1G05660 RT-RP | GGCAACCTTCGTGAGTAGGA | Quantitative real-time PCR |
| CASP3 RT-LP | GATGGCGACAACGGAAGAGA | Quantitative real-time PCR |
| CASP3 RT-RP | GTAGCCACCCACGATTGAGT | Quantitative real-time PCR |
| EXP18 RT-LP | TGTATCGCAAGTGCCTGGTT | Quantitative real-time PCR |
| EXP18 RT-RP | CGCATGCATAACCCTCGTTG | Quantitative real-time PCR |
| XTH12 RT-LP | CTTGGACAGCTGAAGTGGGT | Quantitative real-time PCR |
| XTH12 RT-RP | TGAGGTTGCATTCAGTGGGT | Quantitative real-time PCR |
| PBS3 RT-LP | CCAGTTATGCTGACACCTCAACATT | Quantitative real-time PCR |
| PBS3 RT-RP | TCAAGCGACTCCTCCATTACCAA | Quantitative real-time PCR |
| ICS1 RT-LP | CAGGTACGAGCTTTTGTCCA | Quantitative real-time PCR |
| ICS1 RT-RP | GAGAACCCCTTATCCCCCAT | Quantitative real-time PCR |
| CBP60G RT-LP | GAGAGGAGATACAGCGTAGTCTTCA | Quantitative real-time PCR |
| CBP60G RT-RP | CGATCACAAGCGGAGAACCATC | Quantitative real-time PCR |
| EPS1 RT-LP | CGACGAGGCTGATCAAAAGA | Quantitative real-time PCR |
| EPS1 RT-RP | TTAGCCGTTGCCTCATGTTT | Quantitative real-time PCR |
| RAB18 RT-LP | GGCTTGGGAGGAATGCTT | Quantitative real-time PCR |
| RAB18 RT-RP | TTGATCTTTTGTGTTATTCCCTTCT | Quantitative real-time PCR |
| LTI30 RT-LP | GGGACTAACACGGCTTATGG | Quantitative real-time PCR |
| LTI30 RT-RP | CTAGTGATGACCACCGGGAA | Quantitative real-time PCR |
| TZF4 RT-LP | CTAACTCACCTCAGGCGAAC | Quantitative real-time PCR |
| TZF4 RT-RP | AGATCAGGGTCACCACAAGA | Quantitative real-time PCR |
| DAA1 RT-LP | ACGCAAAGTCTCGGTAATGA | Quantitative real-time PCR |
| DAA1 RT-RP | GCGGCGGATATGAGAGATTT | Quantitative real-time PCR |
| NADP-ME1 RT-LP | GTGACACCTCTCTGCATCCT | Quantitative real-time PCR |
| NADP-ME1 RT-RP | TACACATCAACAACGCCACC | Quantitative real-time PCR |
| AT1G6850 RT-LP | GTTTCACGGTTCACGCTCTA | Quantitative real-time PCR |
| AT1G6850 RT-RP | CAGAATCGACATCAGCCGAA | Quantitative real-time PCR |
| LEA4-5 RT-LP | CAGAAGAGAGAAACGCGTCA | Quantitative real-time PCR |
| LEA4-5 RT-RP | CCAGTTCCAGTGTTCCTTCC | Quantitative real-time PCR |
| KIN1 RT-LP | ATGCCTTCCAAGCCGGTCAGAC | Quantitative real-time PCR |
| KIN1 RT-RP | CCGGTCTTGTCCTTCACGAAGT | Quantitative real-time PCR |
| ABCG6 RT-LP | AGAAGTCTCTCCGTGTCCAA | Quantitative real-time PCR |
| ABCG6 RT-RP | TCGAGGAAAAGCAGAATCGG | Quantitative real-time PCR |
| Actin RT-LP | ATGACTCAGATCATGTTTGAGACC | Quantitative real-time PCR |
| Actin RT-RP | TCAGTAAGGTCACGACCAGCAA | Quantitative real-time PCR |
| SALK_094400 LP | AGAATATACTGGCGGAGGAGG | Genotyping |
| SALK_094400 RP | AATCGGAAGATTCGTACCGTC | Genotyping |
| GABI_017A08 LP | AGGTTGAAGAATGTGACGGTGT | Genotyping |
| GABI_017A08 RP | TCTTAAGTAATGGAAACGACATCG​ | Genotyping |
| SALK_013968 LP | AATAGGAGAGCTCGGAGTTGG | Genotyping |
| SALK_013968 RP | ACGCTGACATTGCTGGTTATC | Genotyping |
| *aba2-1* LP | GATGACTCGCGGTACCTAA | Genotyping |
| *aba2-1* RP | AAAACCACAATAAGCTCCACAG | Genotyping |
| *sid2-2* LP | CTCAACCTCCGTCGTTTTCC | Genotyping |
| *sid2-2* RP | ATTGCTCCGATTTGCTGGTC | Genotyping |
| LB1.3 | ATTTTGCCGATTTCGGAAC | Genotyping |
| LB1 (GABI) | ATATTGACCATCATACTCATTGC | Genotyping |
| JAZ1 LP | GGGGACAAGTTTGTACAAAAAAGCAGGCTTCGAAGGAGATAGAACCATGTCGAGTTCTATGGAATGTTCT | Transgenic plants |
| JAZ1 RP | GGGGACCACTTTGTACAAGAAAGCTGGGTCTATTTCAGCTGCTAAACCGAG | Transgenic plants |
| JAZ9 LP | GGGGACAAGTTTGTACAAAAAAGCAGGCTTCGAAGGAGATAGAACCATGGAAAGAGATTTTCTGGGTTT | Transgenic plants |
| JAZ9 RP | GGGGACCACTTTGTACAAGAAAGCTGGGTCTGTAGGAGAAGTAGAAGAGTAATTCATT | Transgenic plants |
