# Supplementary figures and images for: The LRXs-RALFs-FER module controls plant growth and salt stress responses by modulating multiple plant hormones
Source: Natl Sci Rev. 2020 Jun 30;8(1):nwaa149. doi: 10.1093/nsr/nwaa149 (PMC8288382; doi:10.1093/nsr/nwaa149)

Figure S1

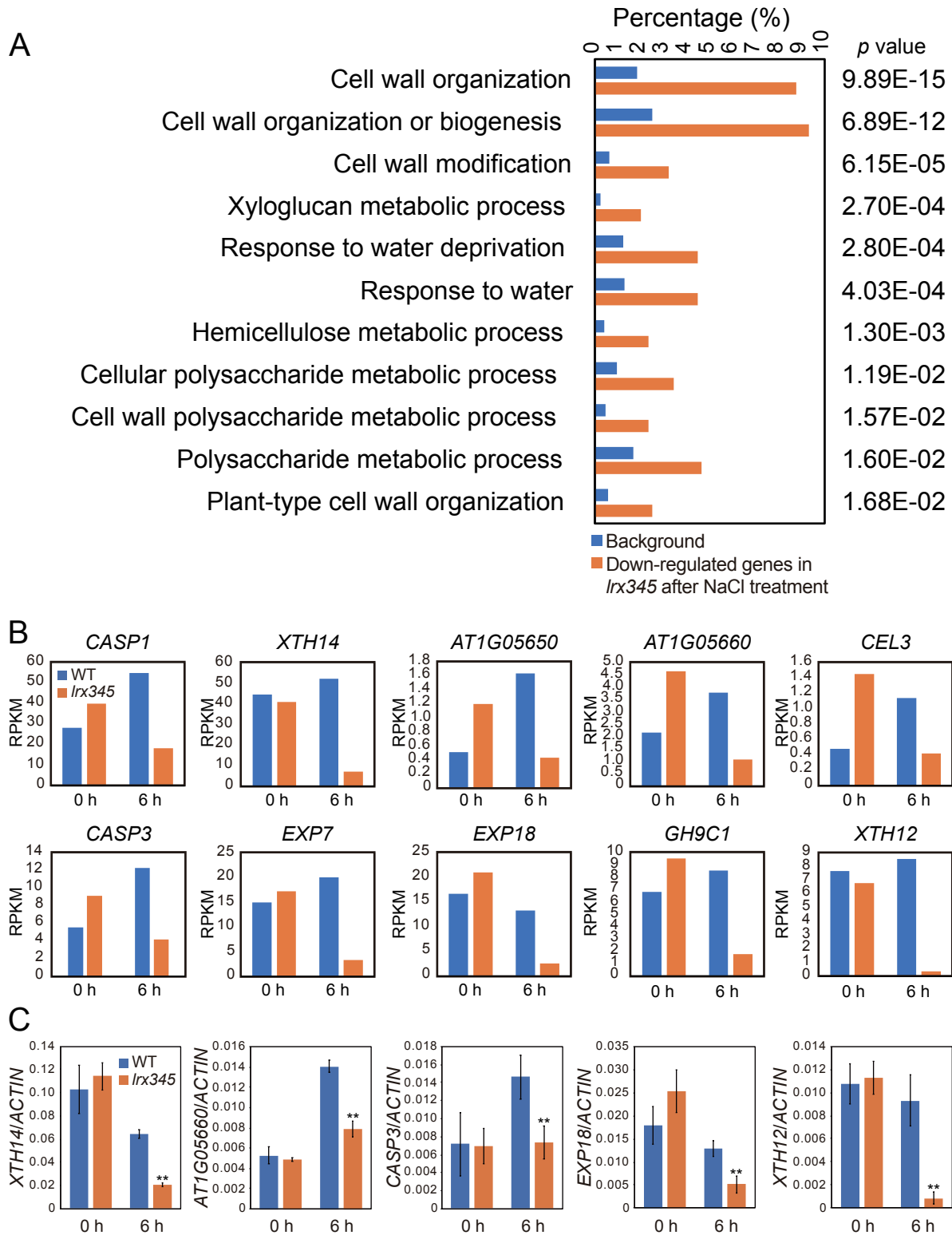

Supplement: nwaa149_Supplemental_Files [file nwaa149_supplemental_files.zip › SFigure 1.pdf]

Figure S10

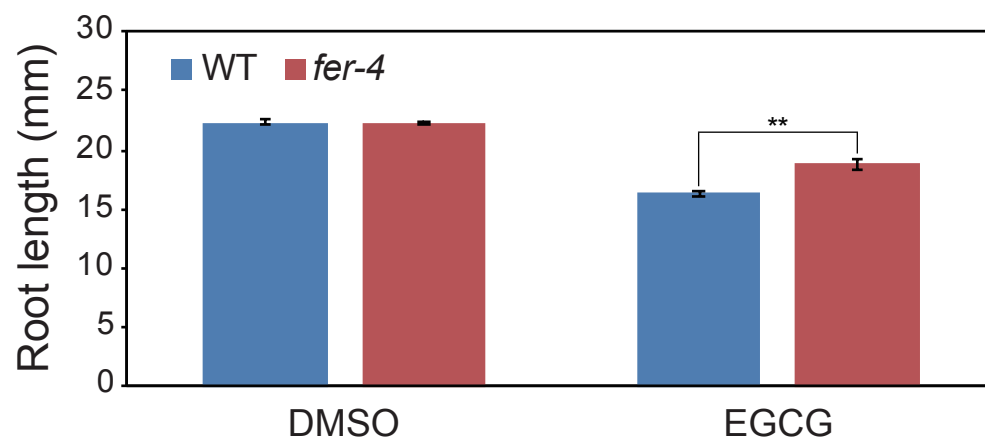

Supplement: nwaa149_Supplemental_Files [file nwaa149_supplemental_files.zip › SFigure 10.pdf]

Figure S11

A

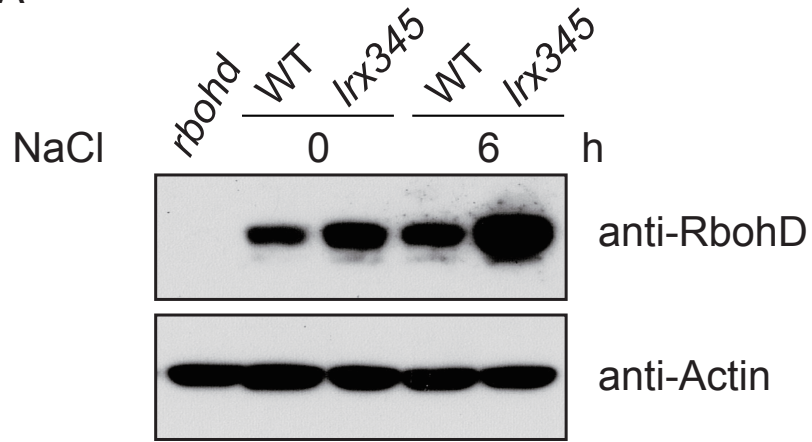

B

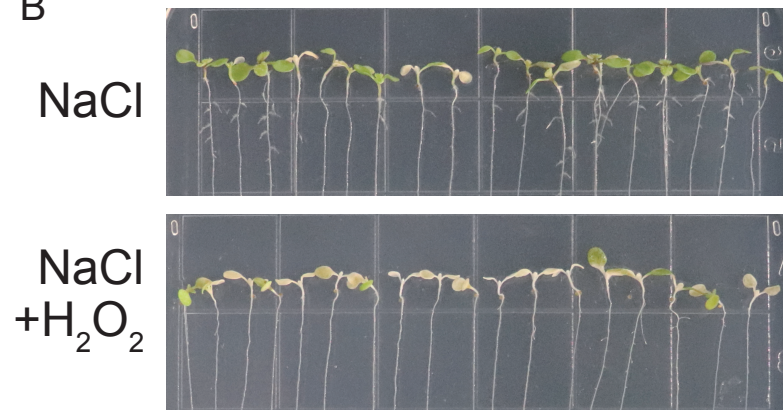

C

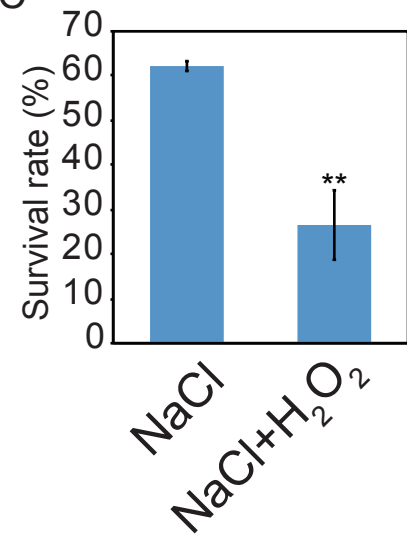

Supplement: nwaa149_Supplemental_Files [file nwaa149_supplemental_files.zip › SFigure 11.pdf]

Figure S12

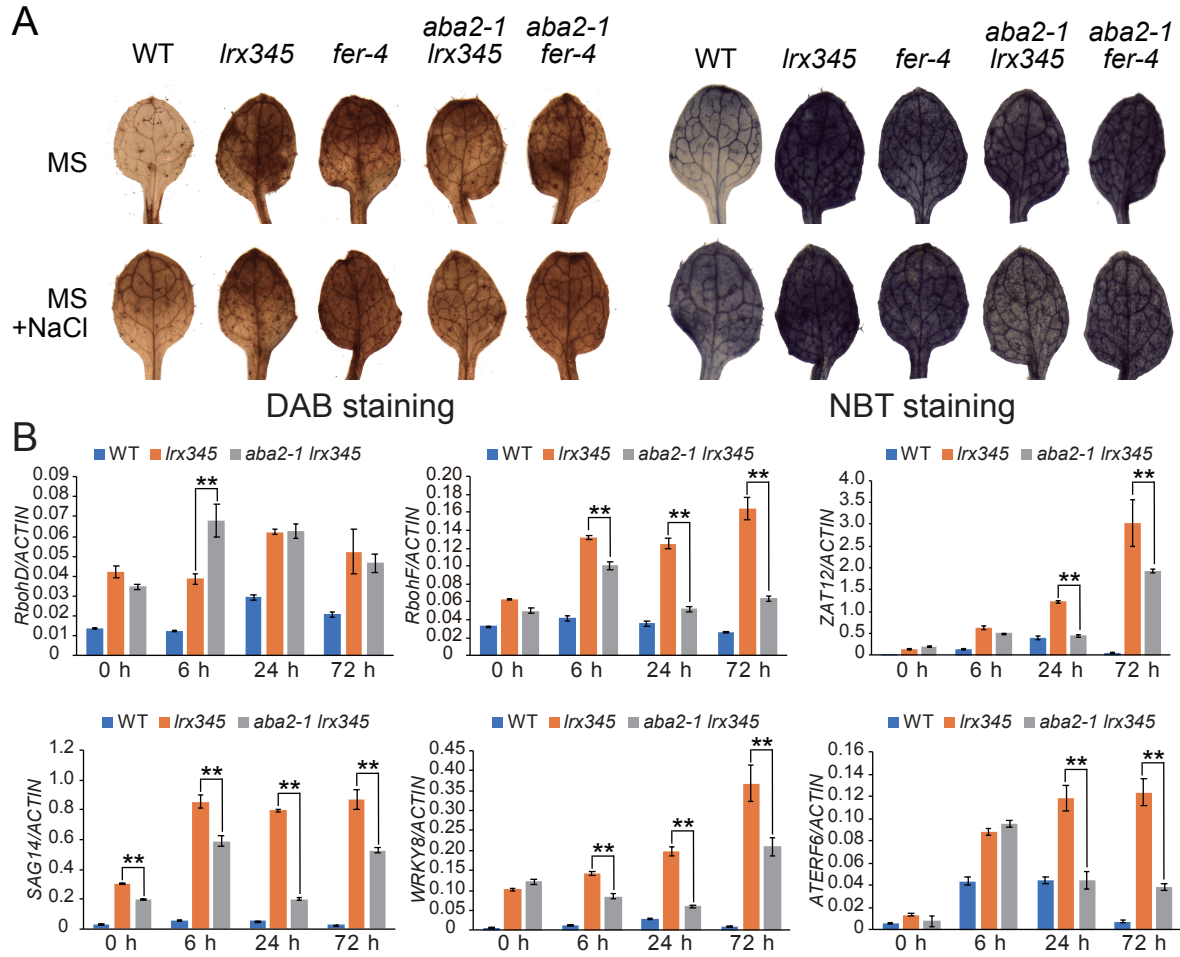

Supplement: nwaa149_Supplemental_Files [file nwaa149_supplemental_files.zip › SFigure 12.pdf]

Figure S2

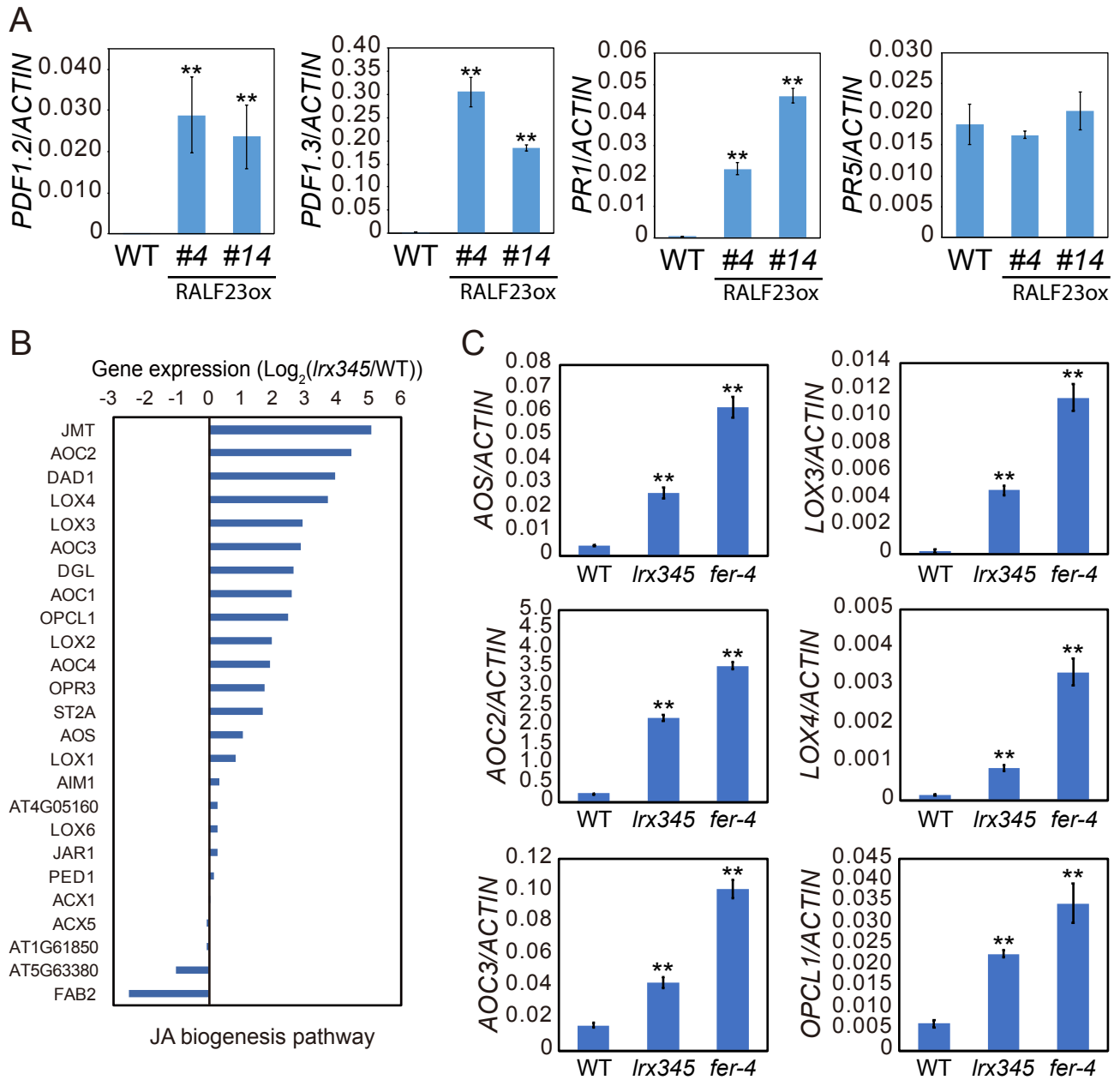

Supplement: nwaa149_Supplemental_Files [file nwaa149_supplemental_files.zip › SFigure 2.pdf]

Figure S3

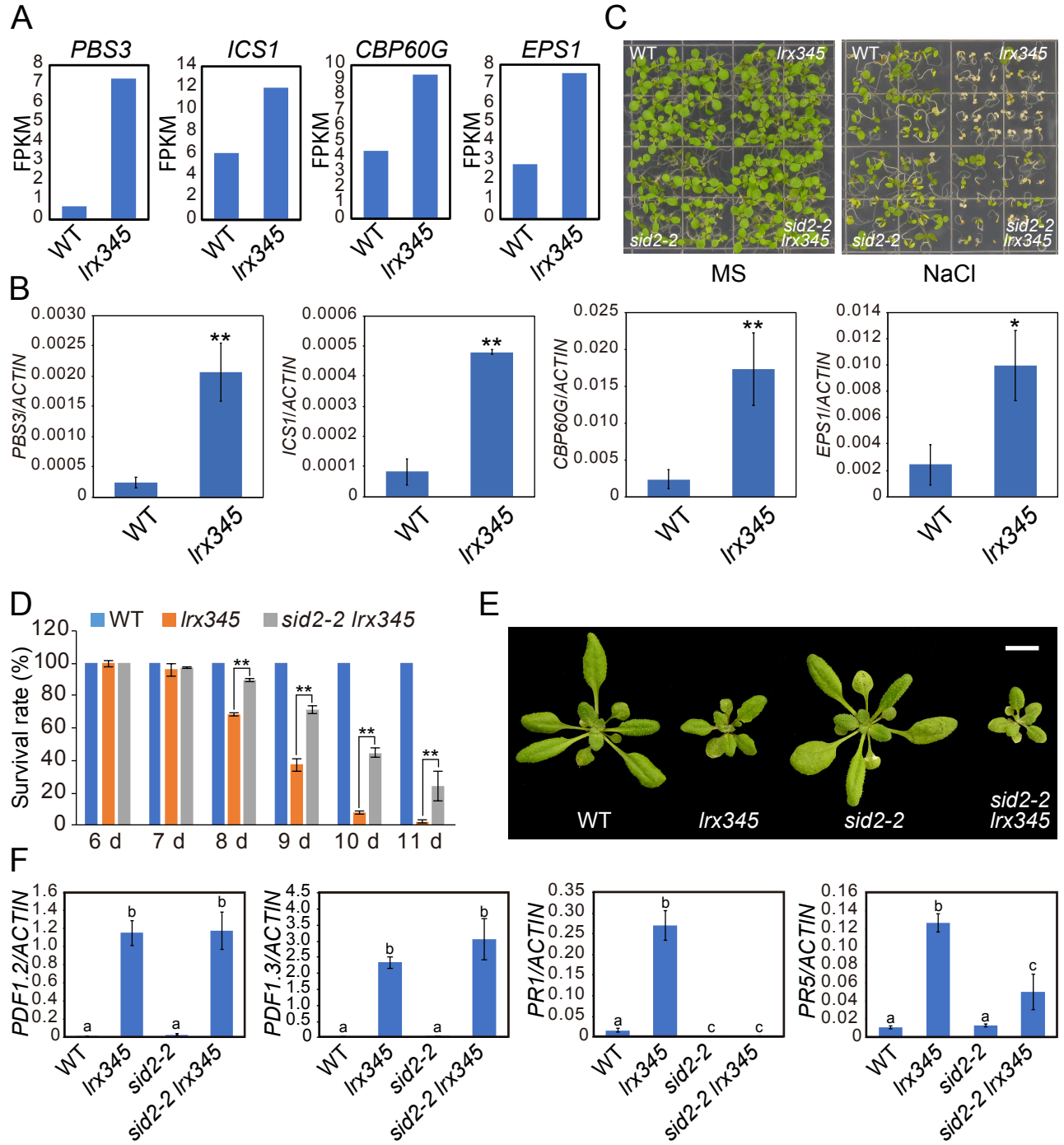

Supplement: nwaa149_Supplemental_Files [file nwaa149_supplemental_files.zip › SFigure 3.pdf]

Figure S4

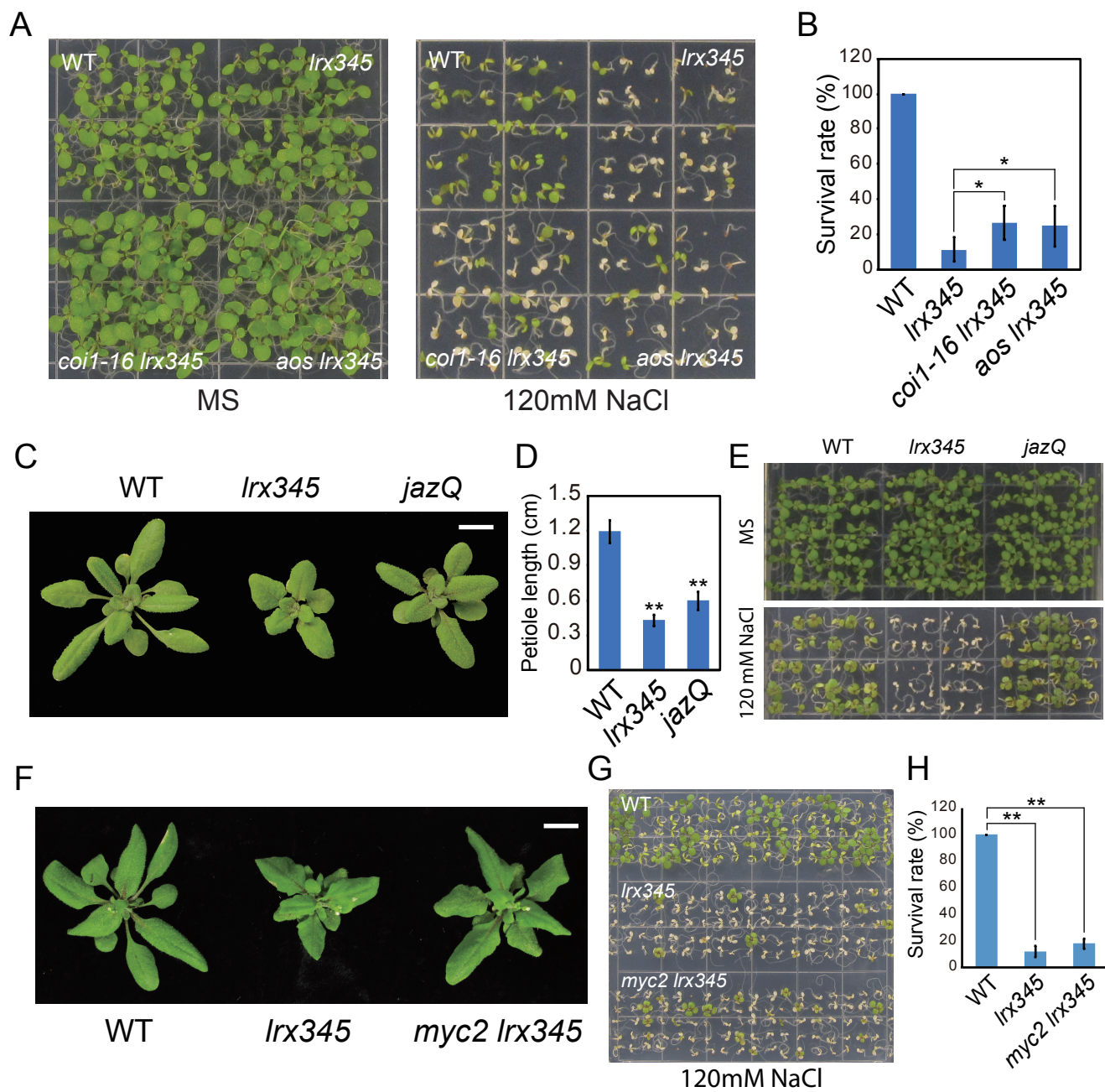

Supplement: nwaa149_Supplemental_Files [file nwaa149_supplemental_files.zip › SFigure 4.pdf]

Figure S6

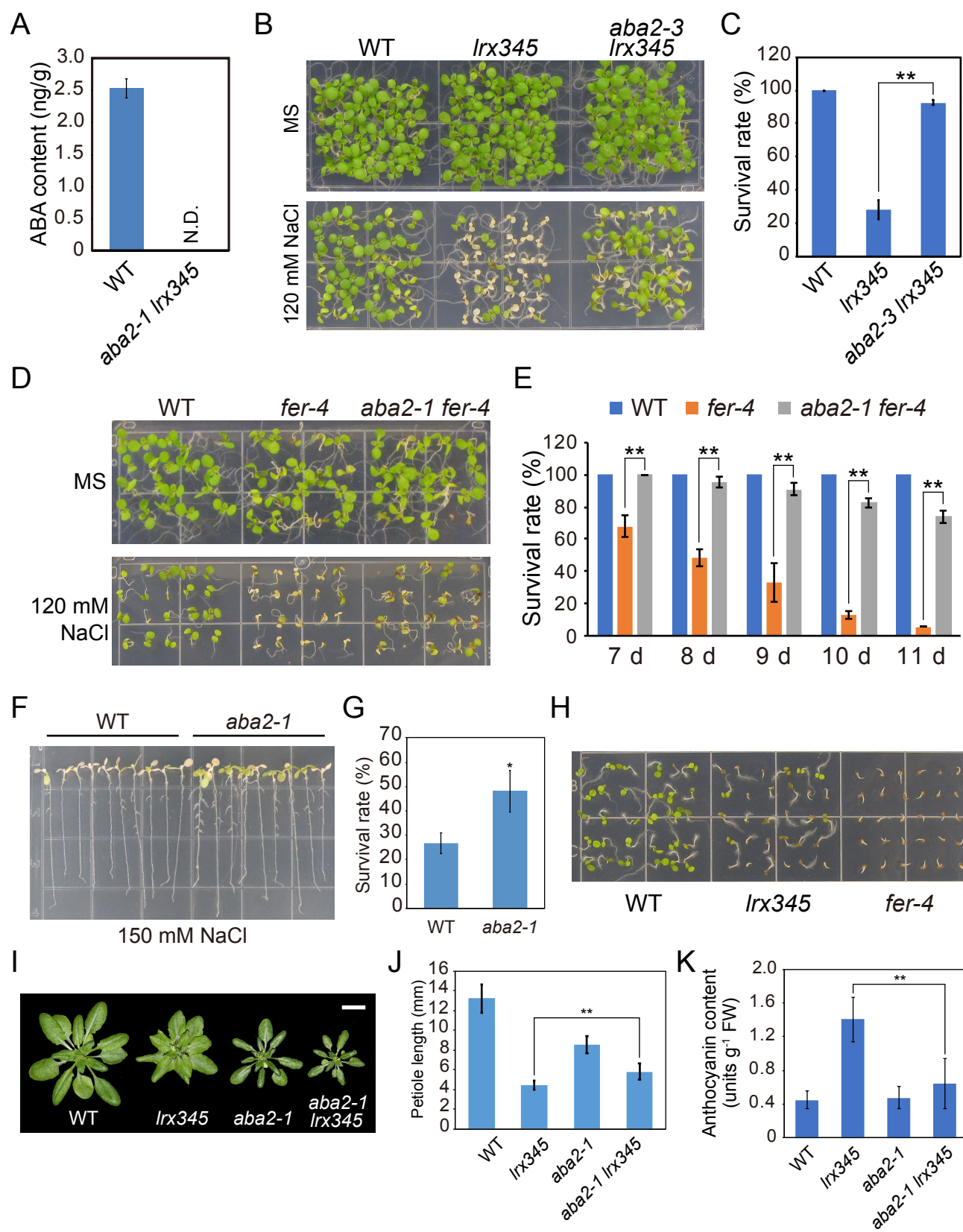

Supplement: nwaa149_Supplemental_Files [file nwaa149_supplemental_files.zip › SFigure 6.pdf]

Figure S7

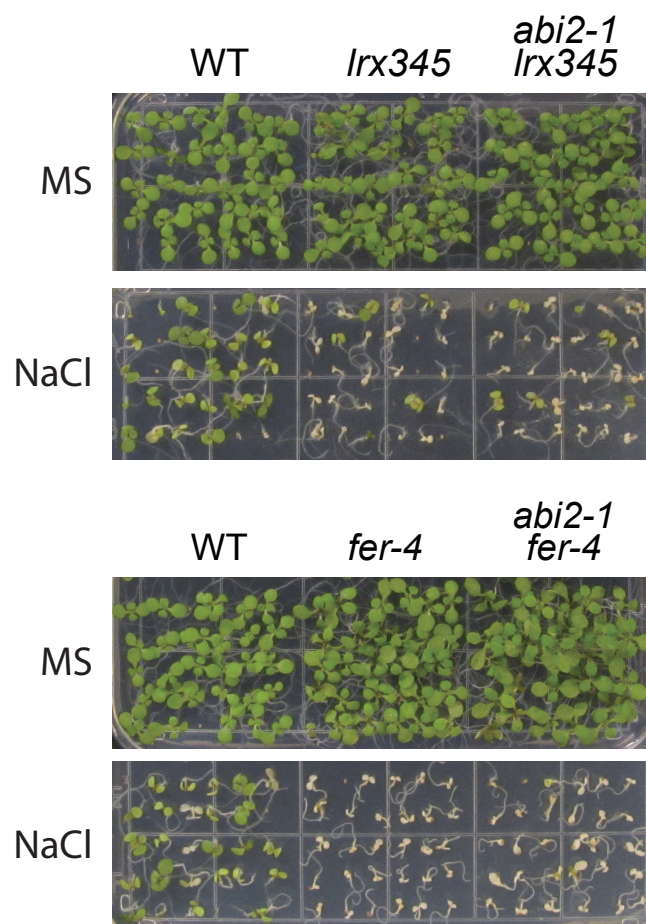

Supplement: nwaa149_Supplemental_Files [file nwaa149_supplemental_files.zip › SFigure 7.pdf]

Figure S8

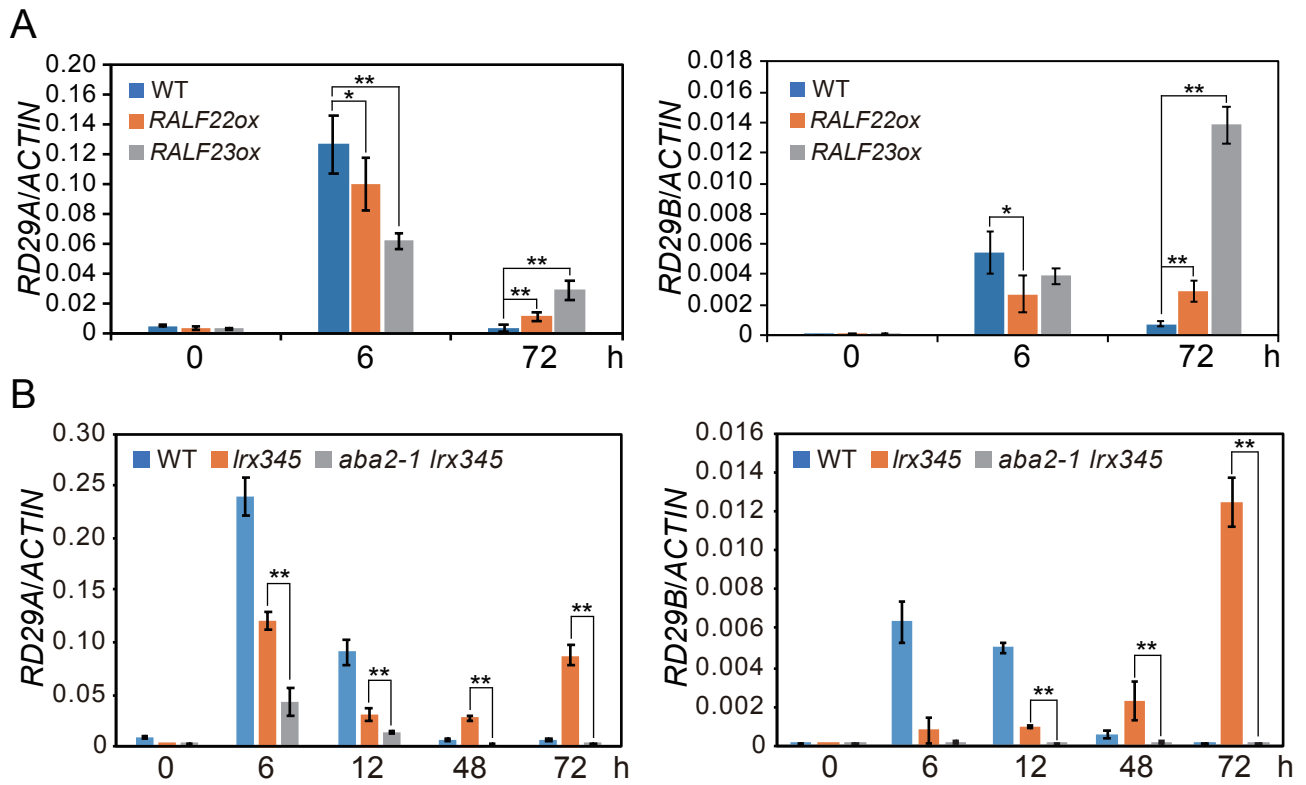

Supplement: nwaa149_Supplemental_Files [file nwaa149_supplemental_files.zip › SFigure 8.pdf]

Figure S9

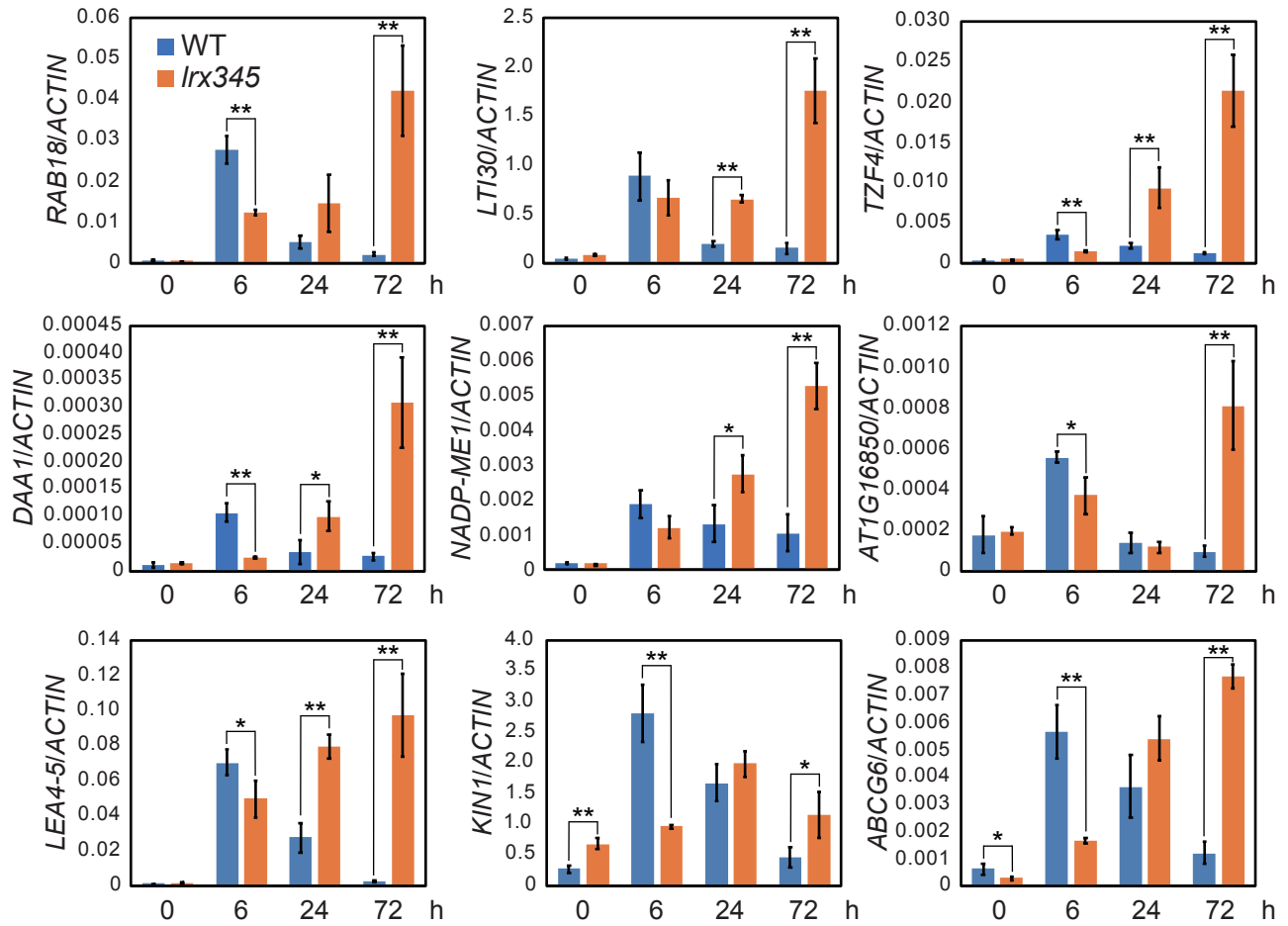

Supplement: nwaa149_Supplemental_Files [file nwaa149_supplemental_files.zip › SFigure 9.pdf]
